# Supplementary material for: Pathogenic mutations of human phosphorylation sites affect protein–protein interactions
Source: Nat Commun. 2024 Apr 11;15:3146. doi: 10.1038/s41467-024-46794-8 (PMC11009412; doi:10.1038/s41467-024-46794-8)
Supplement: Supplementary file 1 — Supplementary Information [file 41467_2024_46794_MOESM1_ESM.pdf]

Supplementary Information

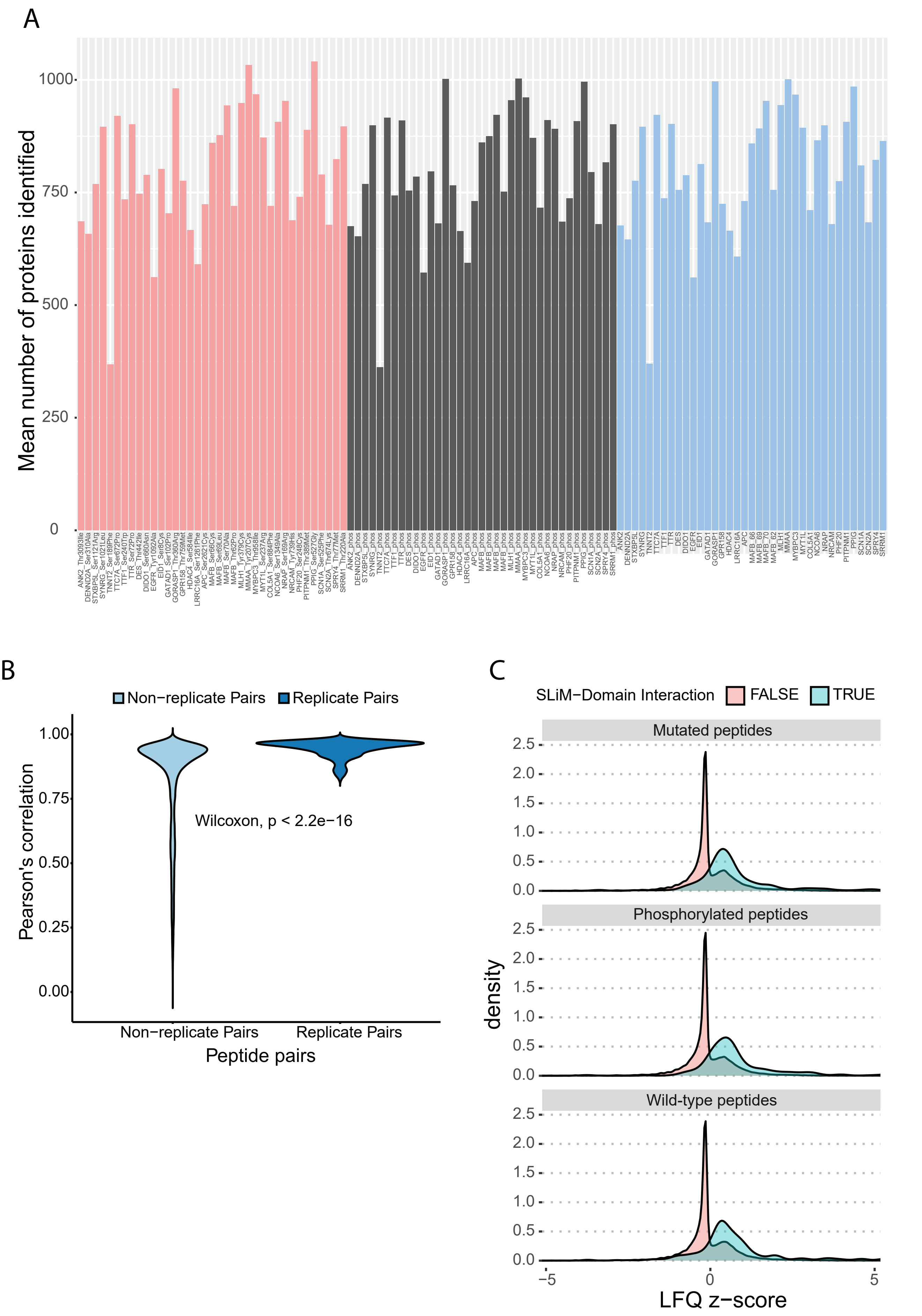

Supplementary Figure 1: A) Bar plot showing the mean total number of proteins identified in each pull-down (n=3). B) Replicate pairs show a high correlation that changes between 0.75 to 1 while non-replicate pairs correlate less and with a higher variability. C) Analysis of the identified interactors, after categorizing them based on their association with Short Linear Motif (SLiM)-Domain pairs, reveals that interactions explained by SLiM-Domain pairs show higher LFQ values. LFQ values were standardized (z-scored) for each protein across all peptides.

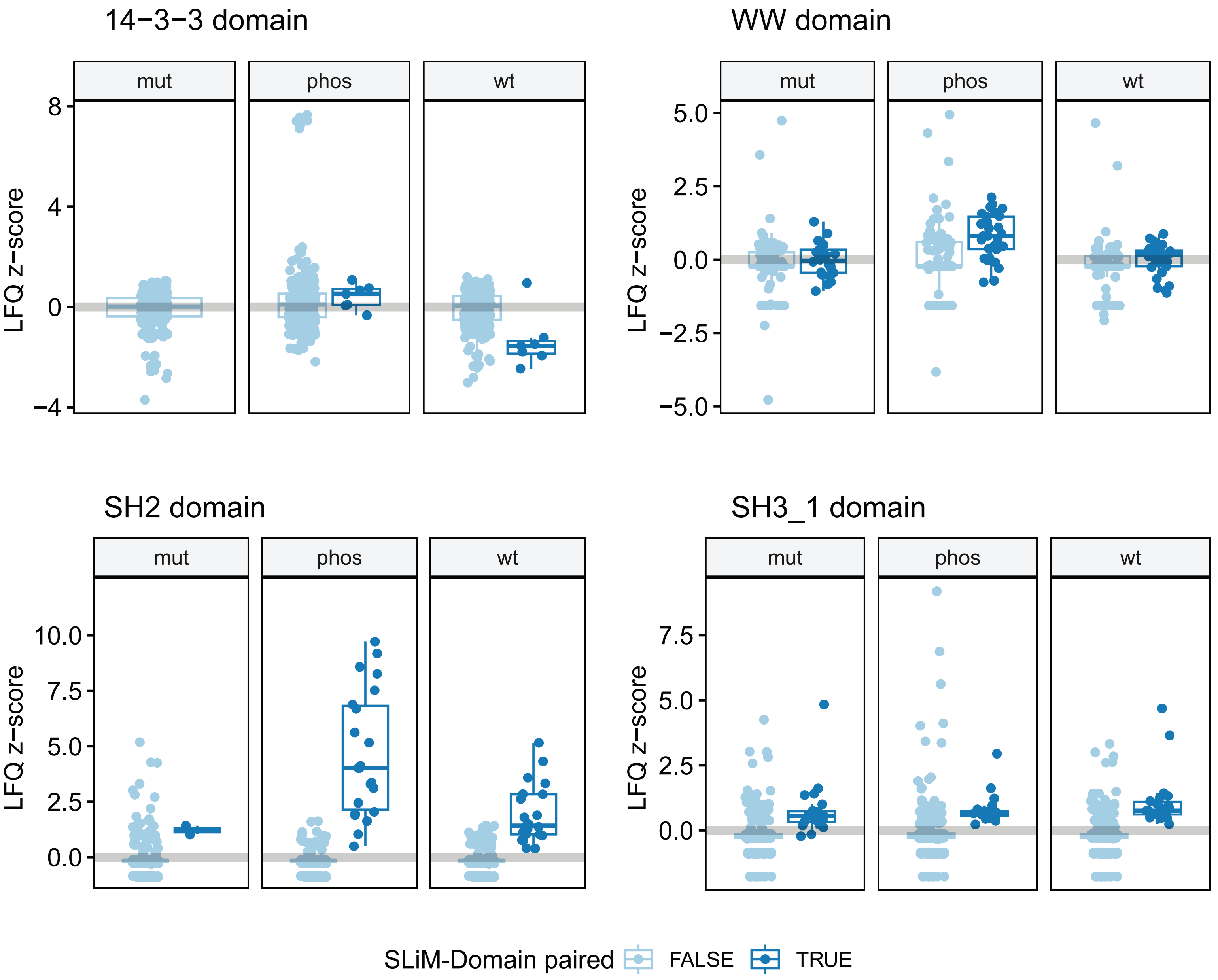

Supplementary Figure 2: LFQ distribution of four SLiM-Domain pairs observed in the PRISMA screen. The observed interactions from each peptide form (mut, phos, wt) are separated as those explained by SLiM-Domain pairs (TRUE) and those that are not (FALSE), and the normalized LFQ values were plotted as box plots. It can be observed that 14-3-3 proteins and WW and SH2 domains bind in a phospho-dependent manner. SH3 proteins, on the other hand, show a slight preference for non-phosphorylated peptides (wt, mut). In general SLiM-Domain interactions almost always have higher LFQ values than the background binders (FALSE). This analysis reflects the global SLiM-Domain analysis from Figure S1C.

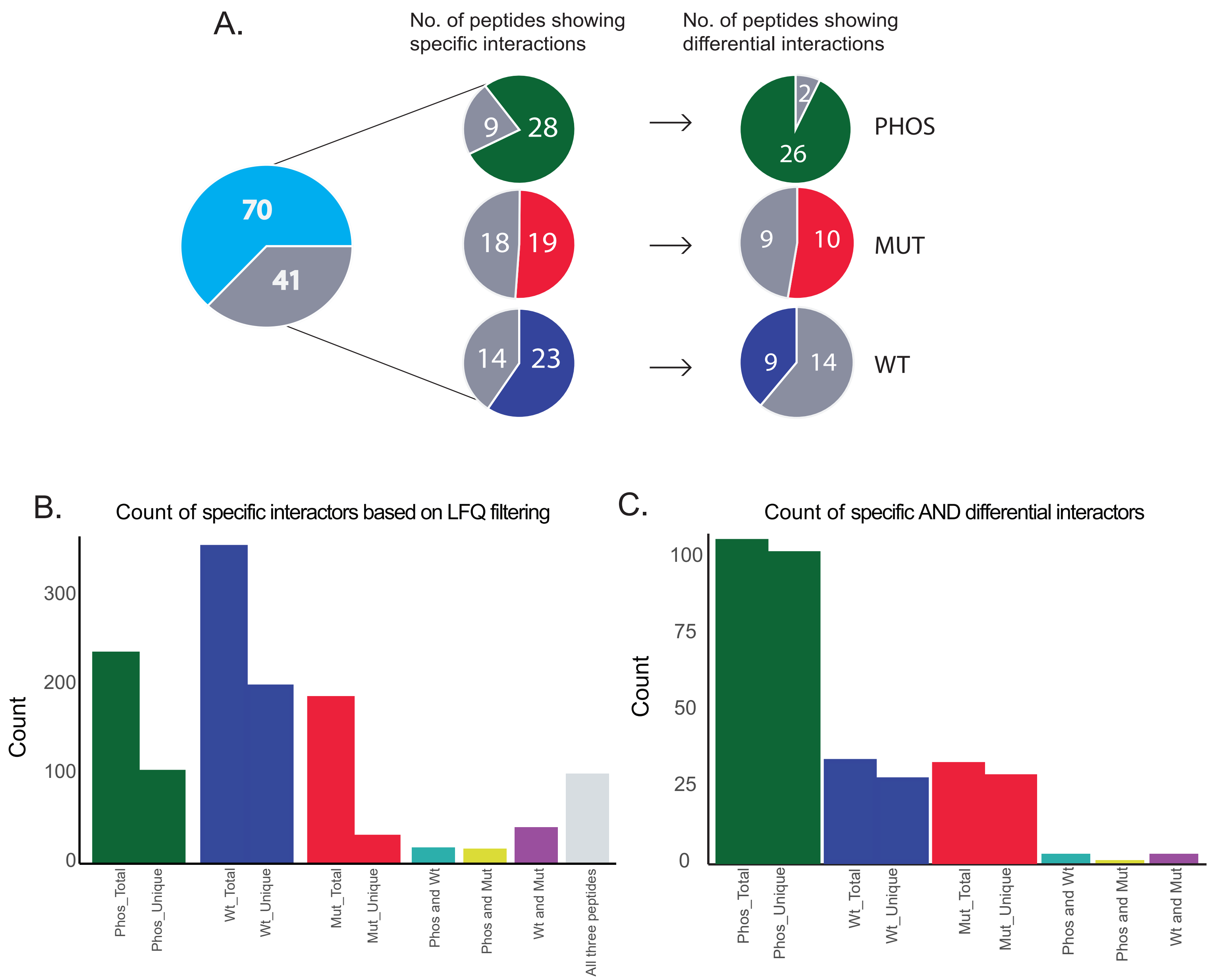

Supplementary Figure 3: A) Pie charts illustrating the number of peptides that exhibit specific or/and differential interactors. B) Bar plot illustrating the total number of proteins identified as significant based on LFQ filtering (Fold change >2 and p values < 0.005) for each pull-down. The y-axis represents the number of proteins, while the x-axis displays the type of interaction. The green bar indicates the number of specific interactors for phosphorylated peptides, with Phos_total including proteins specific to phosphorylated and other peptide forms, and Phos_unique representing proteins exclusively interacting only with the phosphorylated peptides. The same applies to Wt (wild-type) and Mut (mutated) peptides. C) Similar to the bar charts in A, but after implementing the SILAC filtering. These proteins have passed the LFQ significance cut-off and also exhibit a significant SILAC ratio (log2FC >1 or < -1).

ANK2: RTPTEEGpTPTSEQNP

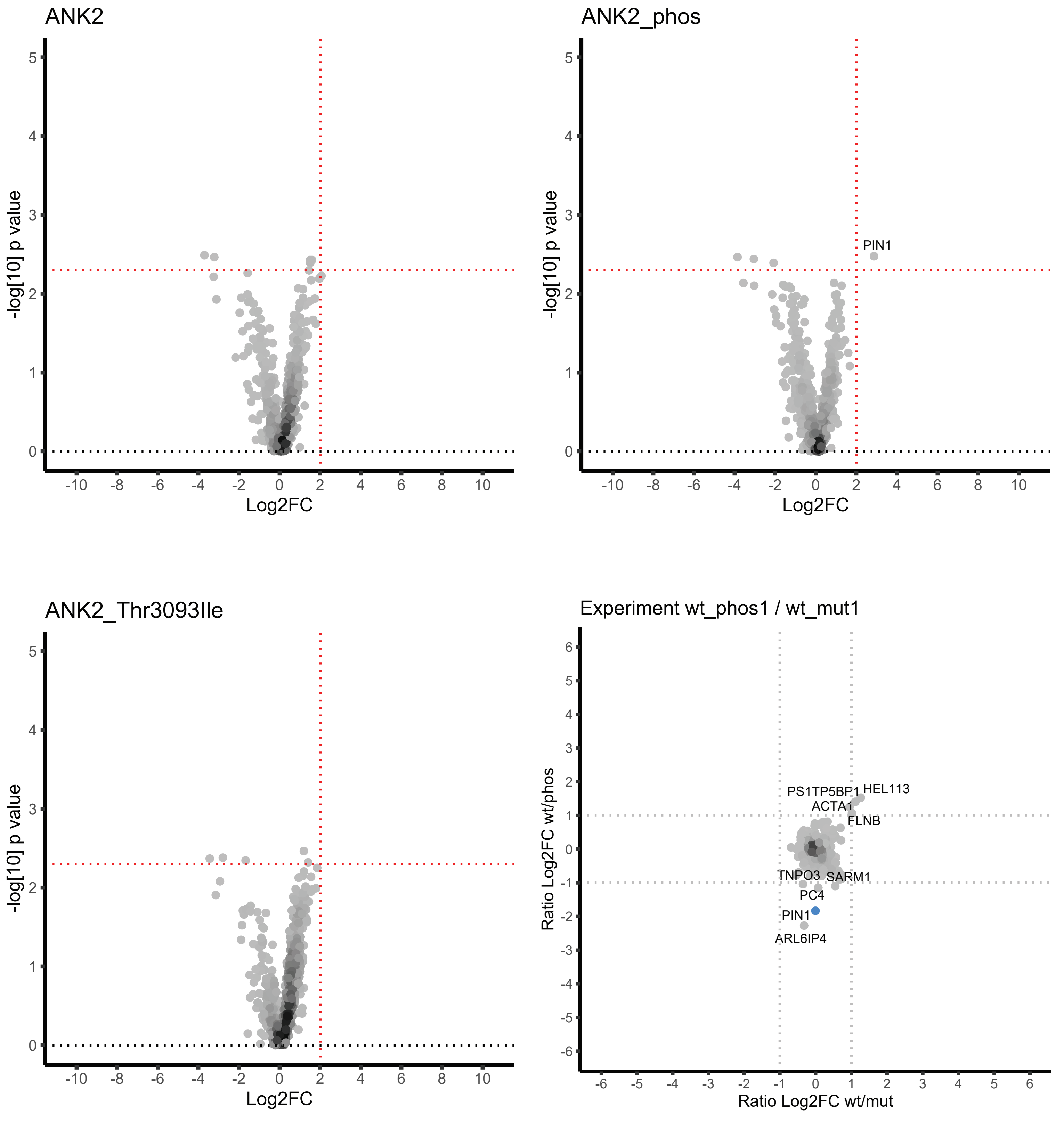

Supplementary Figure 4: Volcano and SILAC scatter plots for the ANK2 peptide candidate: LFQ values were used to perform a Wilcoxon test where proteins identified in one peptide form (triplicates) were compared to all the proteins identified in all other WT and Mutant peptide forms. The calculated p-values and fold changes (Significance:Log2Fold change > 2 and p-value < 0.005) were plotted as Volcano plots (Top and bottom right). The log2 transformed SILAC ratios were plotted on scatter plots with wt/mut ratios on the x-axis and wt/phos ratios on the y-axis (bottom left) whereas the phos/mut ratios are not shown. SILAC ratios reveal differential interactors. To point out LFQ specific interactors in the scatter plots we used colored nodes as follows: Blue; phospho-peptide specific, yellow; wt-peptide specific, red; mut-peptide specific, orange; wt and mut-peptide specific, green; wt and phospho-peptide specific, purple; mut and phospho-peptide specific, and pink; wt, mut and phospho-peptide specific.

APC: KIKENEFpSPTNSTSQ

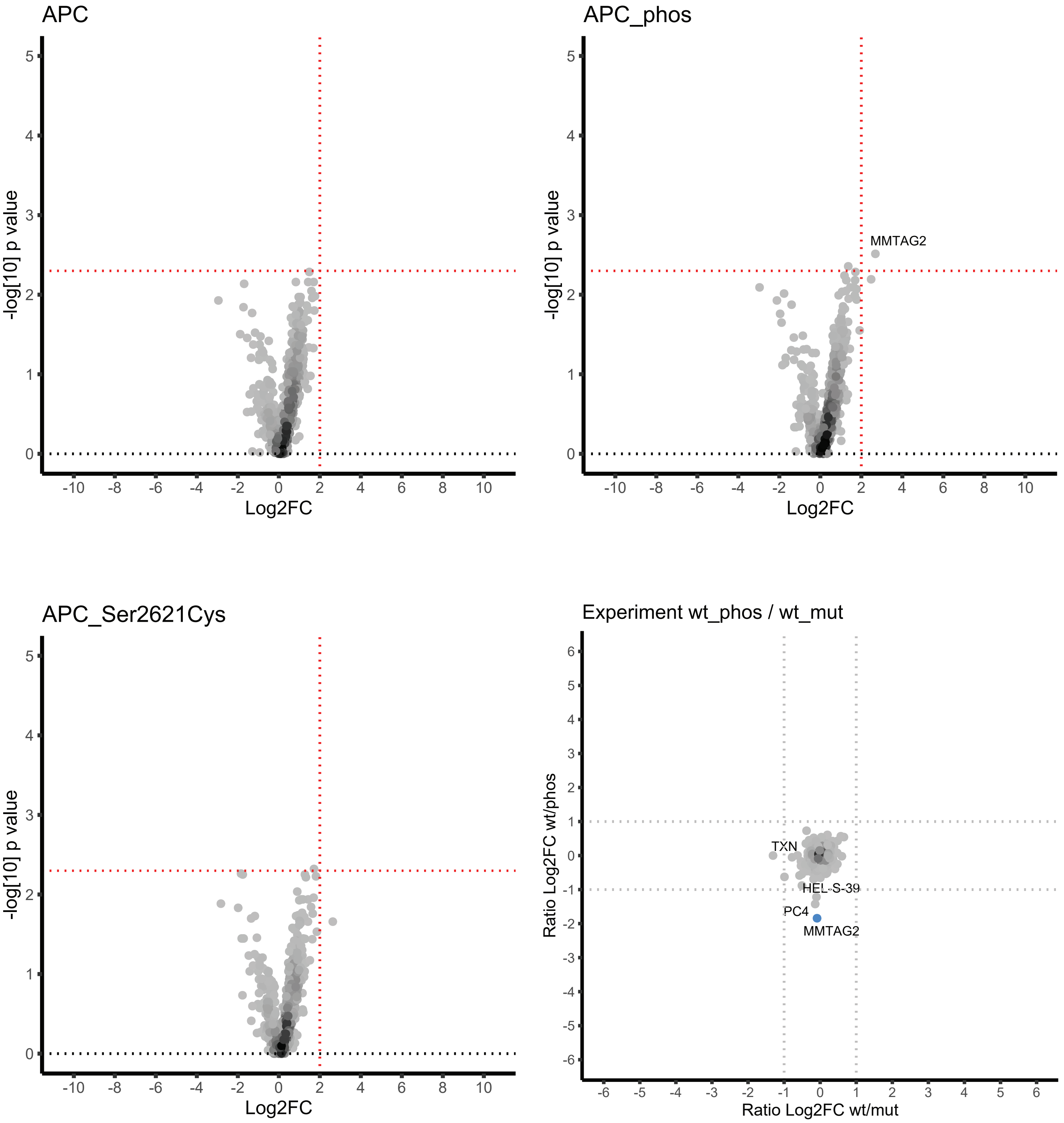

Supplementary Figure 5: Volcano and SILAC scatter plots for the APC peptide candidate: LFQ values were used to perform a Wilcoxon test where proteins identified in one peptide form (triplicates) were compared to all the proteins identified in all other WT and Mutant peptide forms. The calculated p-values and fold changes (Significance:Log2Fold change > 2 and p-value < 0.005) were plotted as Volcano plots (Top and bottom right). The log2 transformed SILAC ratios were plotted on scatter plots with wt/mut ratios on the x-axis and wt/phos ratios on the y-axis (bottom left) whereas the phos/mut ratios are not shown. SILAC ratios reveal differential interactors. To point out LFQ specific interactors in the scatter plots we used colored nodes as follows: Blue; phospho-peptide specific, yellow; wt-peptide specific, red; mut-peptide specific, orange; wt and mut-peptide specific, green; wt and phospho-peptide specific, purple; mut and phospho-peptide specific, and pink; wt, mut and phospho-peptide specific.

COL5A1: GRQGPKGpSIGFPGFP

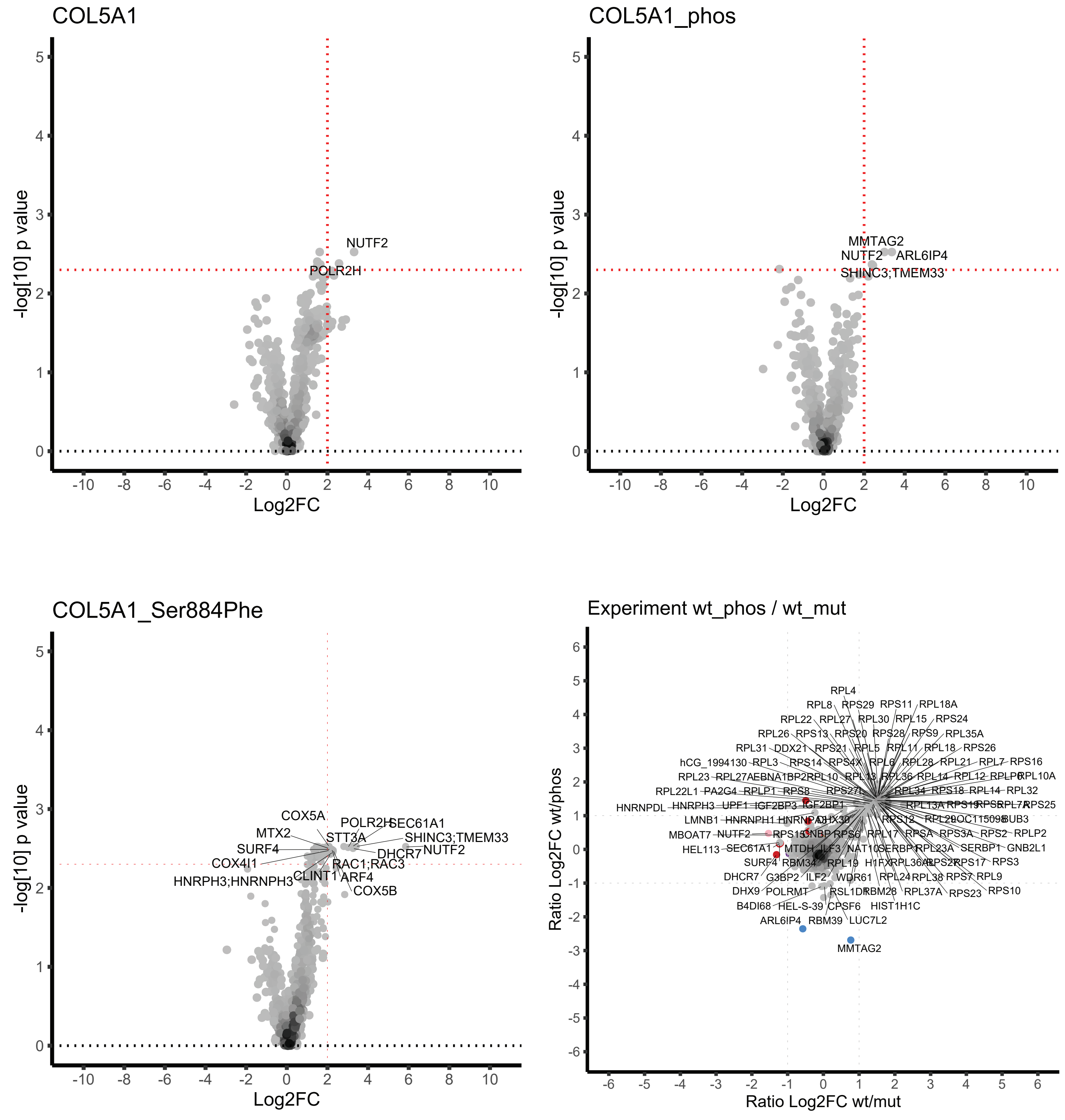

Supplementary Figure 6: Volcano and SILAC scatter plots for the COL5A1 peptide candidate: LFQ values were used to perform a Wilcoxon test where proteins identified in one peptide form (triplicates) were compared to all the proteins identified in all other WT and Mutant peptide forms. The calculated p-values and fold changes (Significance:Log2Fold change > 2 and p-value < 0.005) were plotted as Volcano plots (Top and bottom right). The log2 transformed SILAC ratios were plotted on scatter plots with wt/mut ratios on the x-axis and wt/phos ratios on the y-axis (bottom left) whereas the phos/mut ratios are not shown. SILAC ratios reveal differential interactors. To point out LFQ specific interactors in the scatter plots we used colored nodes as follows: Blue; phospho-peptide specific, yellow; wt-peptide specific, red; mut-peptide specific, orange; wt and mut-peptide specific, green; wt and phospho-peptide specific, purple; mut and phospho-peptide specific, and pink; wt, mut and phospho-peptide specific.

DENND2A: PPPPLPSpSPPPSSVN

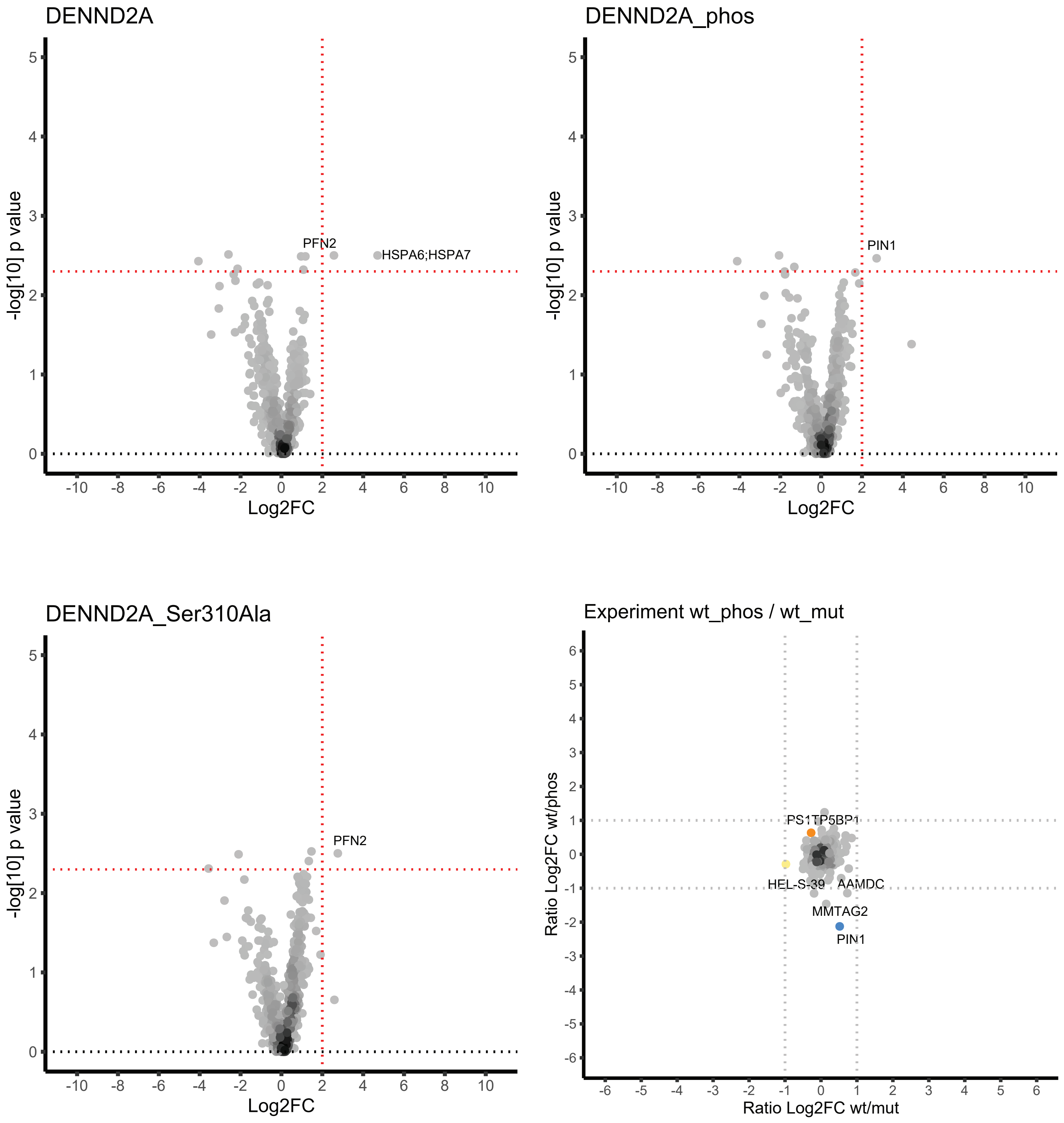

Supplementary Figure 7: Volcano and SILAC scatter plots for the DENND2A peptide candidate: LFQ values were used to perform a Wilcoxon test where proteins identified in one peptide form (triplicates) were compared to all the proteins identified in all other WT and Mutant peptide forms. The calculated p-values and fold changes (Significance:Log2Fold change > 2 and p-value < 0.005) were plotted as Volcano plots (Top and bottom right). The log2 transformed SILAC ratios were plotted on scatter plots with wt/mut ratios on the x-axis and wt/phos ratios on the y-axis (bottom left) whereas the phos/mut ratios are not shown. SILAC ratios reveal differential interactors. To point out LFQ specific interactors in the scatter plots we used colored nodes as follows: Blue; phospho-peptide specific, yellow; wt-peptide specific, red; mut-peptide specific, orange; wt and mut-peptide specific, green; wt and phospho-peptide specific, purple; mut and phospho-peptide specific, and pink; wt, mut and phospho-peptide specific.

DES: QRGSEVHpTKKTVMIK

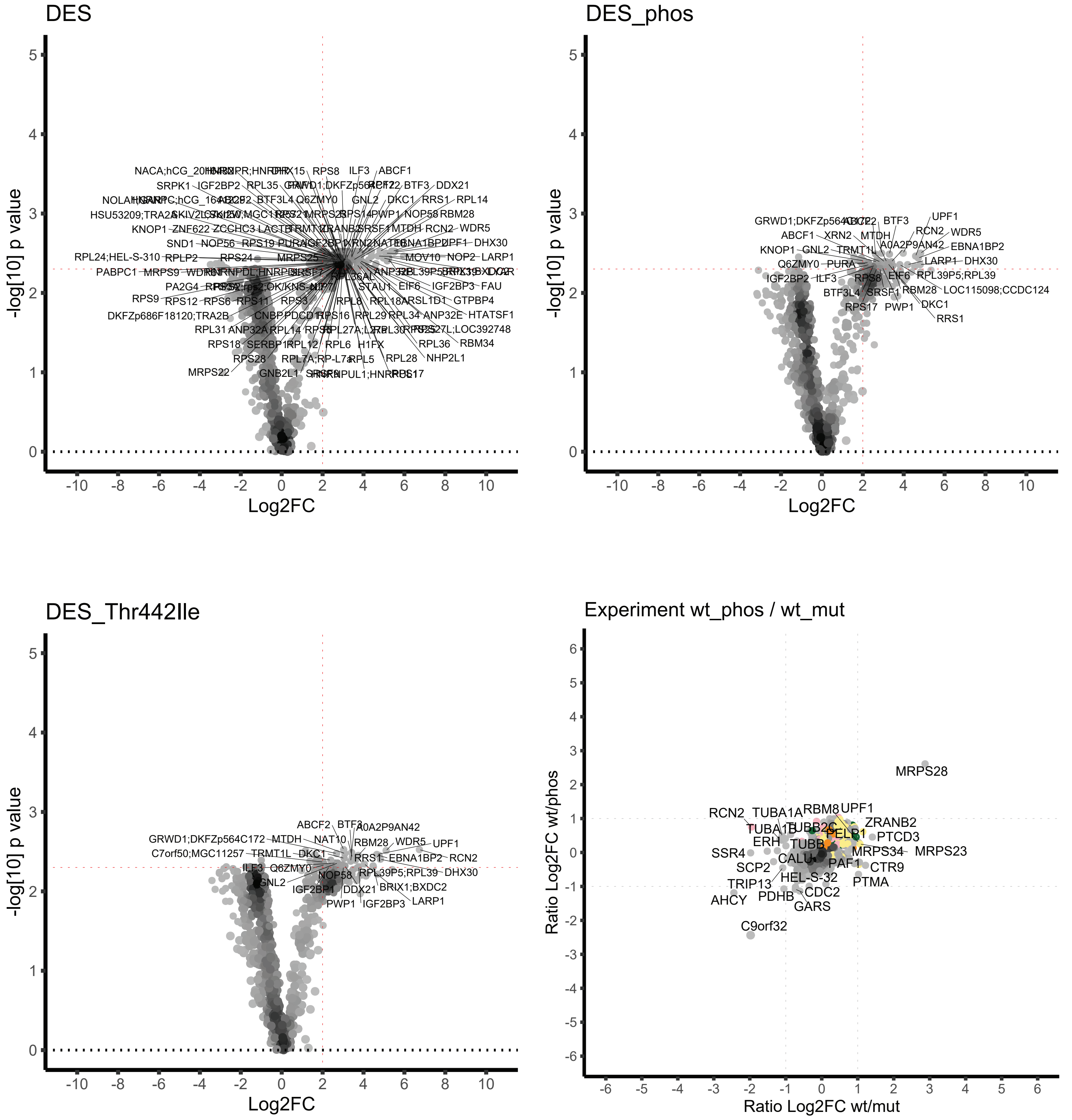

Supplementary Figure 8: Volcano and SILAC scatter plots for the DES peptide candidate: LFQ values were used to perform a Wilcoxon test where proteins identified in one peptide form (triplicates) were compared to all the proteins identified in all other WT and Mutant peptide forms. The calculated p-values and fold changes (Significance:Log2Fold change > 2 and p-value < 0.005) were plotted as Volcano plots (Top and bottom right). The log2 transformed SILAC ratios were plotted on scatter plots with wt/mut ratios on the x-axis and wt/phos ratios on the y-axis (bottom left) whereas the phos/mut ratios are not shown. SILAC ratios reveal differential interactors. To point out LFQ specific interactors in the scatter plots we used colored nodes as follows: Blue; phospho-peptide specific, yellow; wt-peptide specific, red; mut-peptide specific, orange; wt and mut-peptide specific, green; wt and phospho-peptide specific, purple; mut and phospho-peptide specific, and pink; wt, mut and phospho-peptide specific.

DIDO1: PGRLGAMpSAAPSQPN

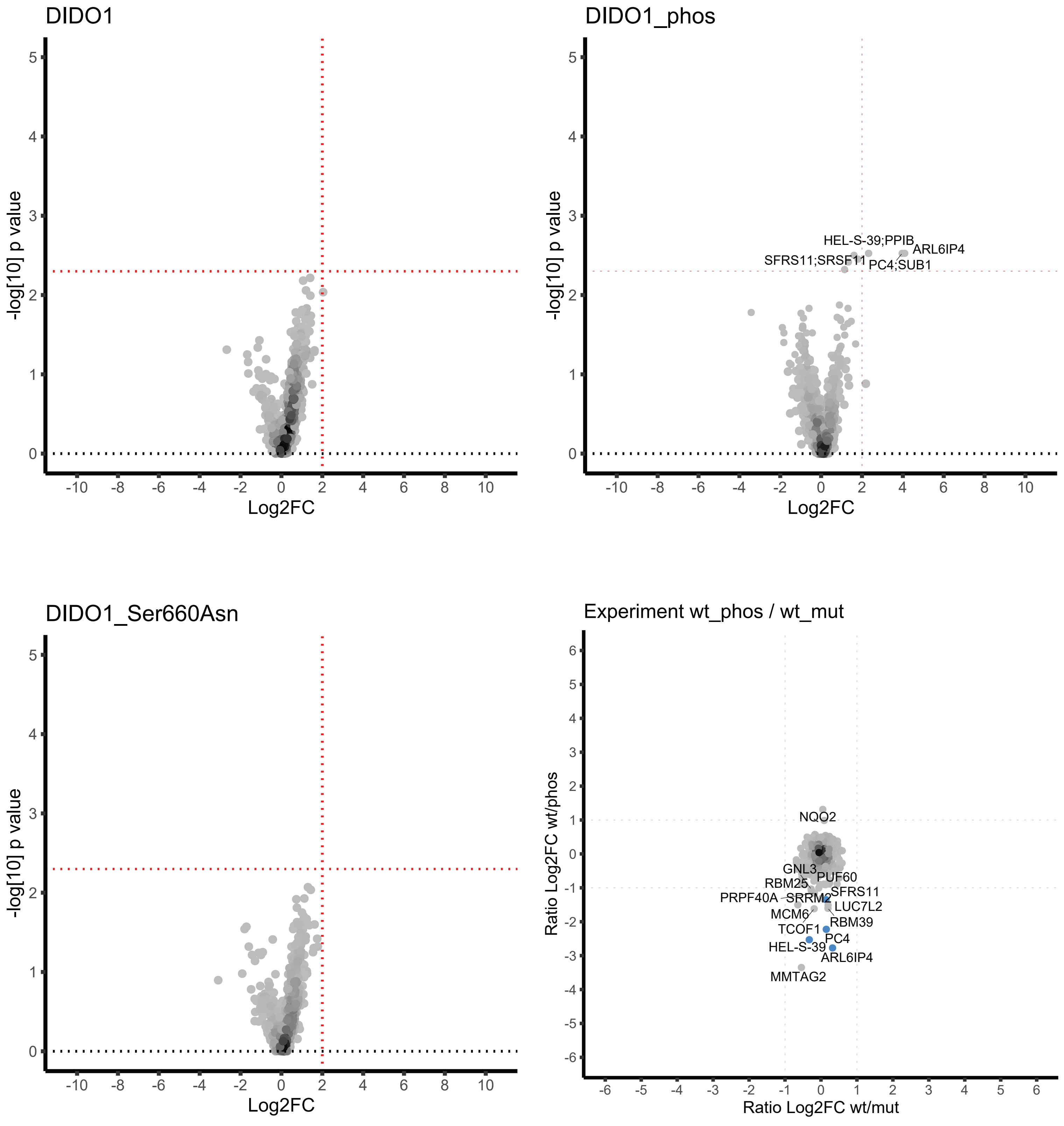

Supplementary Figure 9: Volcano and SILAC scatter plots for the DIDO1 peptide candidate: LFQ values were used to perform a Wilcoxon test where proteins identified in one peptide form (triplicates) were compared to all the proteins identified in all other WT and Mutant peptide forms. The calculated p-values and fold changes (Significance:Log2Fold change > 2 and p-value < 0.005) were plotted as Volcano plots (Top and bottom right). The log2 transformed SILAC ratios were plotted on scatter plots with wt/mut ratios on the x-axis and wt/phos ratios on the y-axis (bottom left) whereas the phos/mut ratios are not shown. SILAC ratios reveal differential interactors. To point out LFQ specific interactors in the scatter plots we used colored nodes as follows: Blue; phospho-peptide specific, yellow; wt-peptide specific, red; mut-peptide specific, orange; wt and mut-peptide specific, green; wt and phospho-peptide specific, purple; mut and phospho-peptide specific, and pink; wt, mut and phospho-peptide specific.

EID1: MSEMAELpSELYEESS

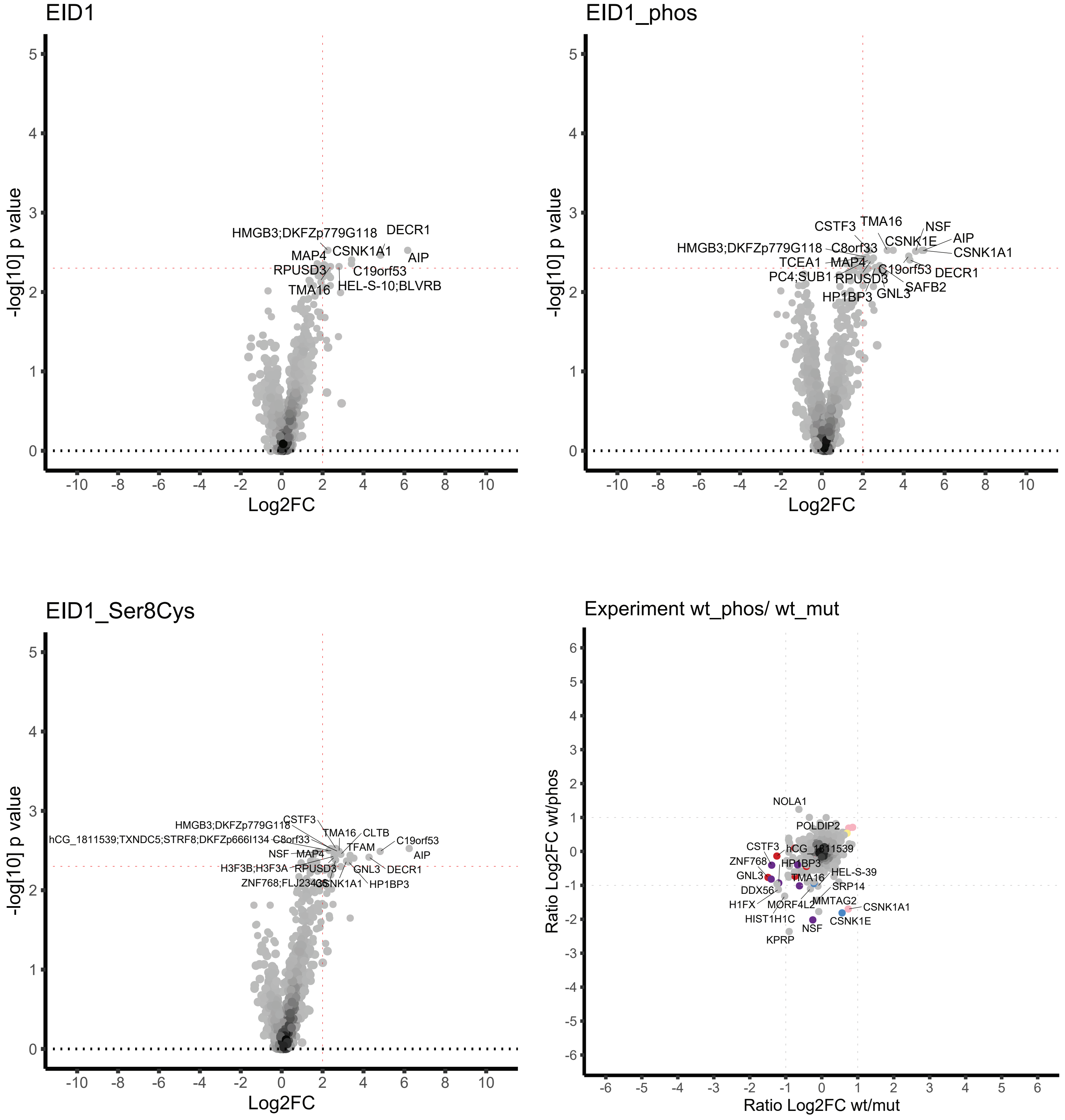

Supplementary Figure 10: Volcano and SILAC scatter plots for the EID1 peptide candidate: LFQ values were used to perform a Wilcoxon test where proteins identified in one peptide form (triplicates) were compared to all the proteins identified in all other WT and Mutant peptide forms. The calculated p-values and fold changes (Significance:Log2Fold change > 2 and p-value < 0.005) were plotted as Volcano plots (Top and bottom right). The log2 transformed SILAC ratios were plotted on scatter plots with wt/mut ratios on the x-axis and wt/phos ratios on the y-axis (bottom left) whereas the phos/mut ratios are not shown. SILAC ratios reveal differential interactors. To point out LFQ specific interactors in the scatter plots we used colored nodes as follows: Blue; phospho-peptide specific, yellow; wt-peptide specific, red; mut-peptide specific, orange; wt and mut-peptide specific, green; wt and phospho-peptide specific, purple; mut and phospho-peptide specific, and pink; wt, mut and phospho-peptide specific.

GATAD1: LRNTKYKpSAPAAEKK

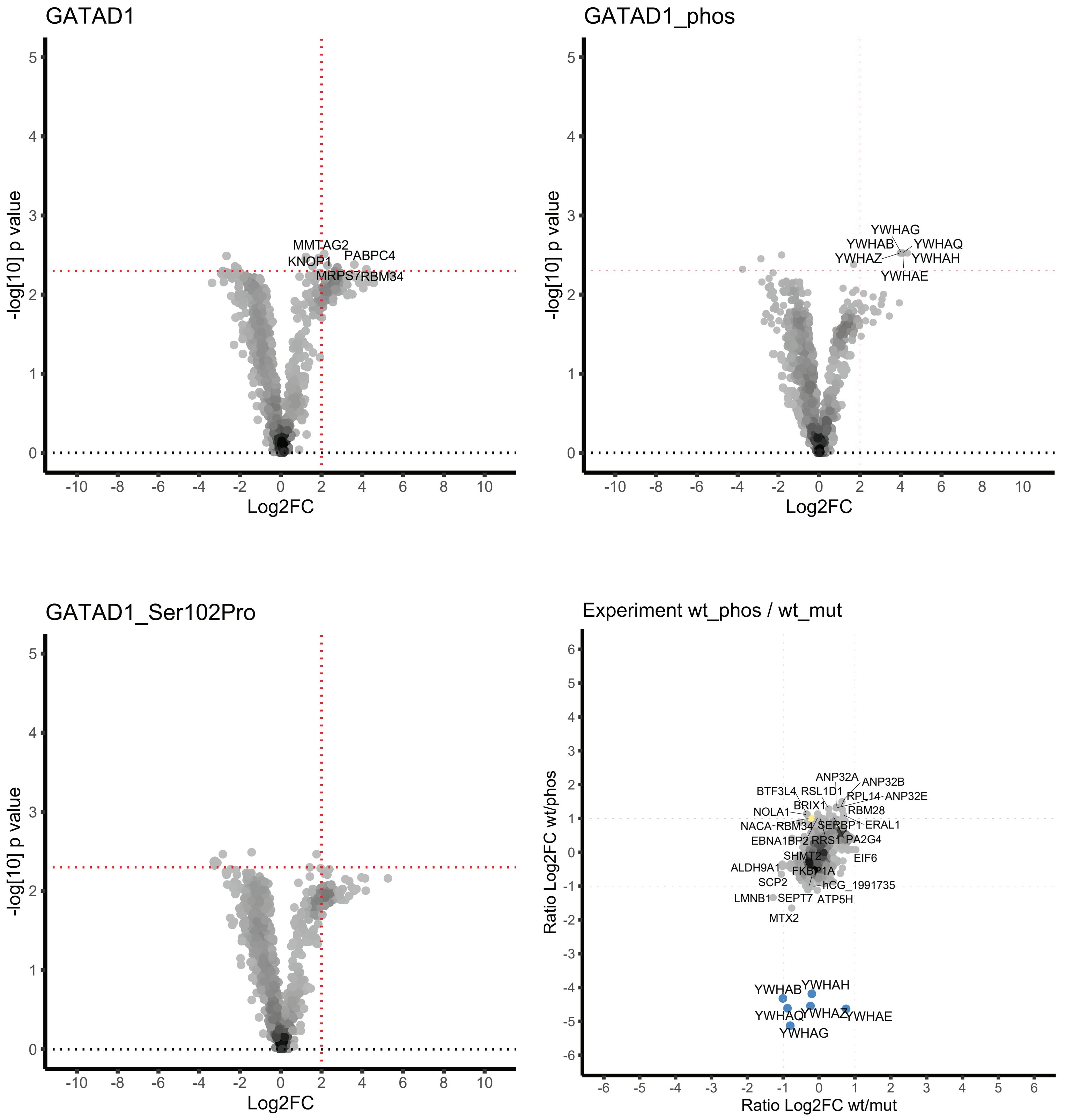

Supplementary Figure 11: Volcano and SILAC scatter plots for the GATAD1 peptide candidate: LFQ values were used to perform a Wilcoxon test where proteins identified in one peptide form (triplicates) were compared to all the proteins identified in all other WT and Mutant peptide forms. The calculated p-values and fold changes (Significance:Log2Fold change > 2 and p-value < 0.005) were plotted as Volcano plots (Top and bottom right). The log2 transformed SILAC ratios were plotted on scatter plots with wt/mut ratios on the x-axis and wt/phos ratios on the y-axis (bottom left) whereas the phos/mut ratios are not shown. SILAC ratios reveal differential interactors. To point out LFQ specific interactors in the scatter plots we used colored nodes as follows: Blue; phospho-peptide specific, yellow; wt-peptide specific, red; mut-peptide specific, orange; wt and mut-peptide specific, green; wt and phospho-peptide specific, purple; mut and phospho-peptide specific, and pink; wt, mut and phospho-peptide specific.

GORASP1: HERGGEApTWSGSEFE

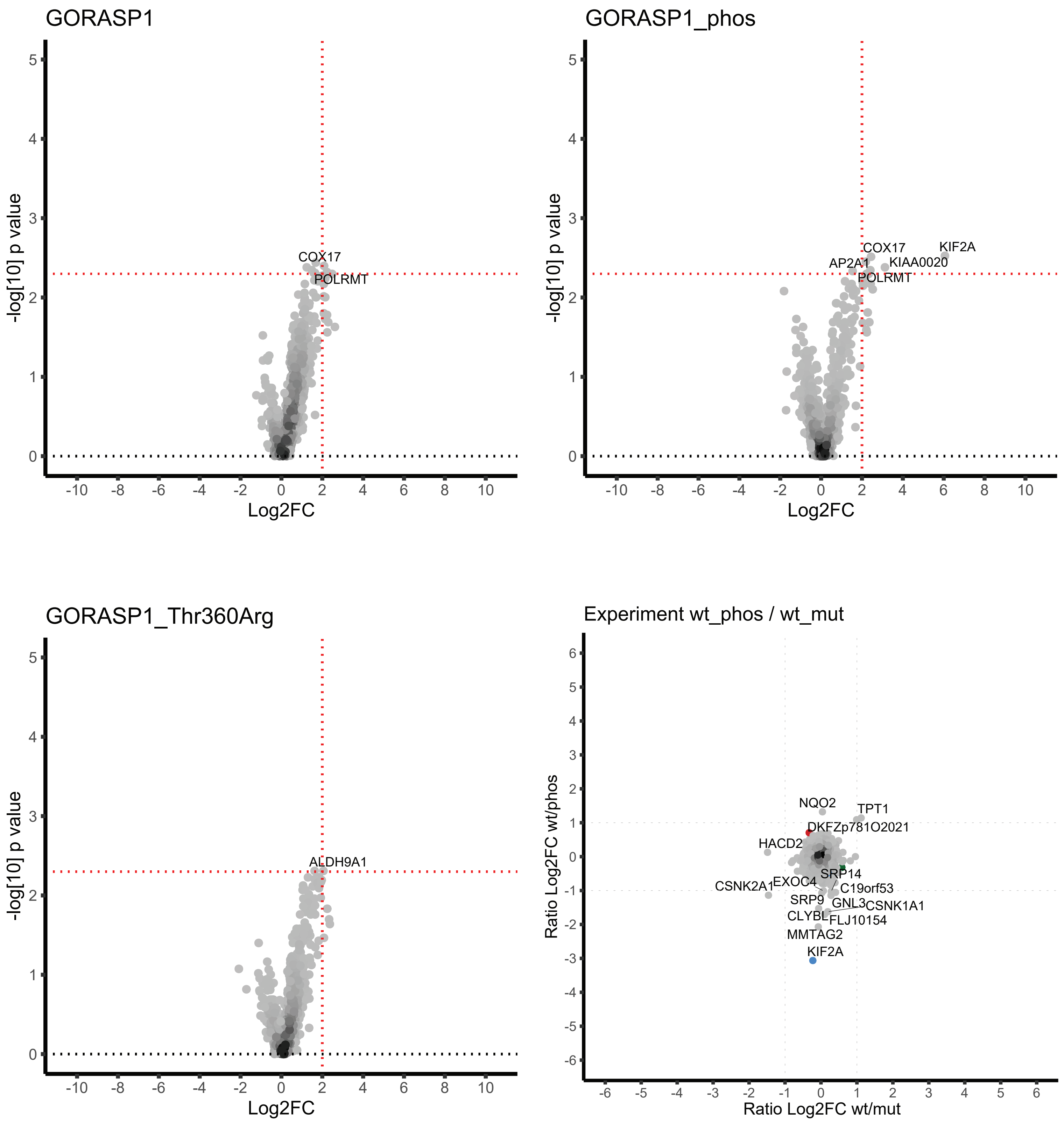

Supplementary Figure 12: Volcano and SILAC scatter plots for the GORASP1 peptide candidate: LFQ values were used to perform a Wilcoxon test where proteins identified in one peptide form (triplicates) were compared to all the proteins identified in all other WT and Mutant peptide forms. The calculated p-values and fold changes (Significance:Log2Fold change > 2 and p-value < 0.005) were plotted as Volcano plots (Top and bottom right). The log2 transformed SILAC ratios were plotted on scatter plots with wt/mut ratios on the x-axis and wt/phos ratios on the y-axis (bottom left) whereas the phos/mut ratios are not shown. SILAC ratios reveal differential interactors. To point out LFQ specific interactors in the scatter plots we used colored nodes as follows: Blue; phospho-peptide specific, yellow; wt-peptide specific, red; mut-peptide specific, orange; wt and mut-peptide specific, green; wt and phospho-peptide specific, purple; mut and phospho-peptide specific, and pink; wt, mut and phospho-peptide specific.

GPR158: RSIMRRIpTEIPETVS

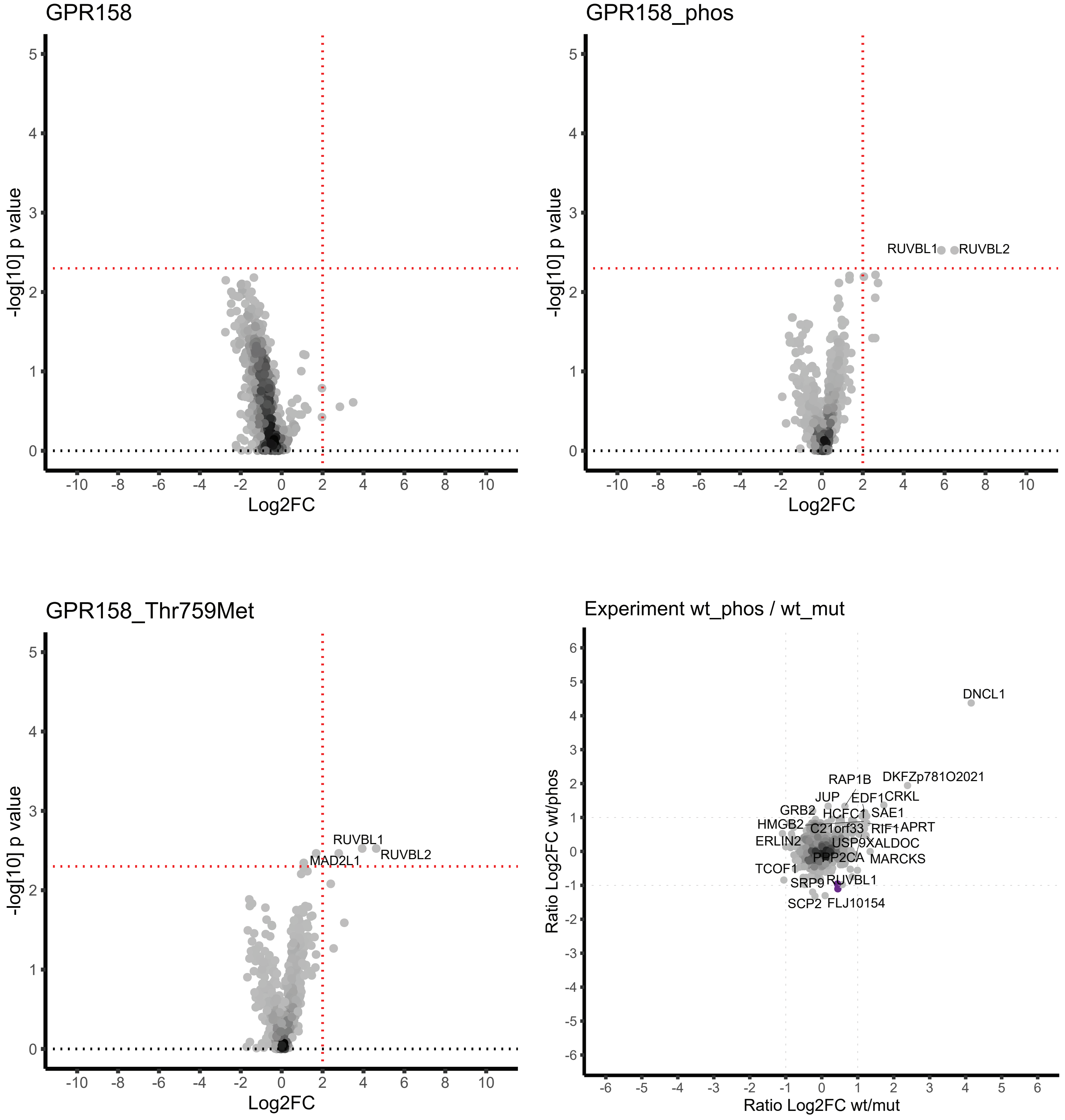

Supplementary Figure 13: Volcano and SILAC scatter plots for the GPR158 peptide candidate: LFQ values were used to perform a Wilcoxon test where proteins identified in one peptide form (triplicates) were compared to all the proteins identified in all other WT and Mutant peptide forms. The calculated p-values and fold changes (Significance:Log2Fold change > 2 and p-value < 0.005) were plotted as Volcano plots (Top and bottom right). The log2 transformed SILAC ratios were plotted on scatter plots with wt/mut ratios on the x-axis and wt/phos ratios on the y-axis (bottom left) whereas the phos/mut ratios are not shown. SILAC ratios reveal differential interactors. To point out LFQ specific interactors in the scatter plots we used colored nodes as follows: Blue; phospho-peptide specific, yellow; wt-peptide specific, red; mut-peptide specific, orange; wt and mut-peptide specific, green; wt and phospho-peptide specific, purple; mut and phospho-peptide specific, and pink; wt, mut and phospho-peptide specific.

HDAC4: EPGQRQPpSEQELLFR

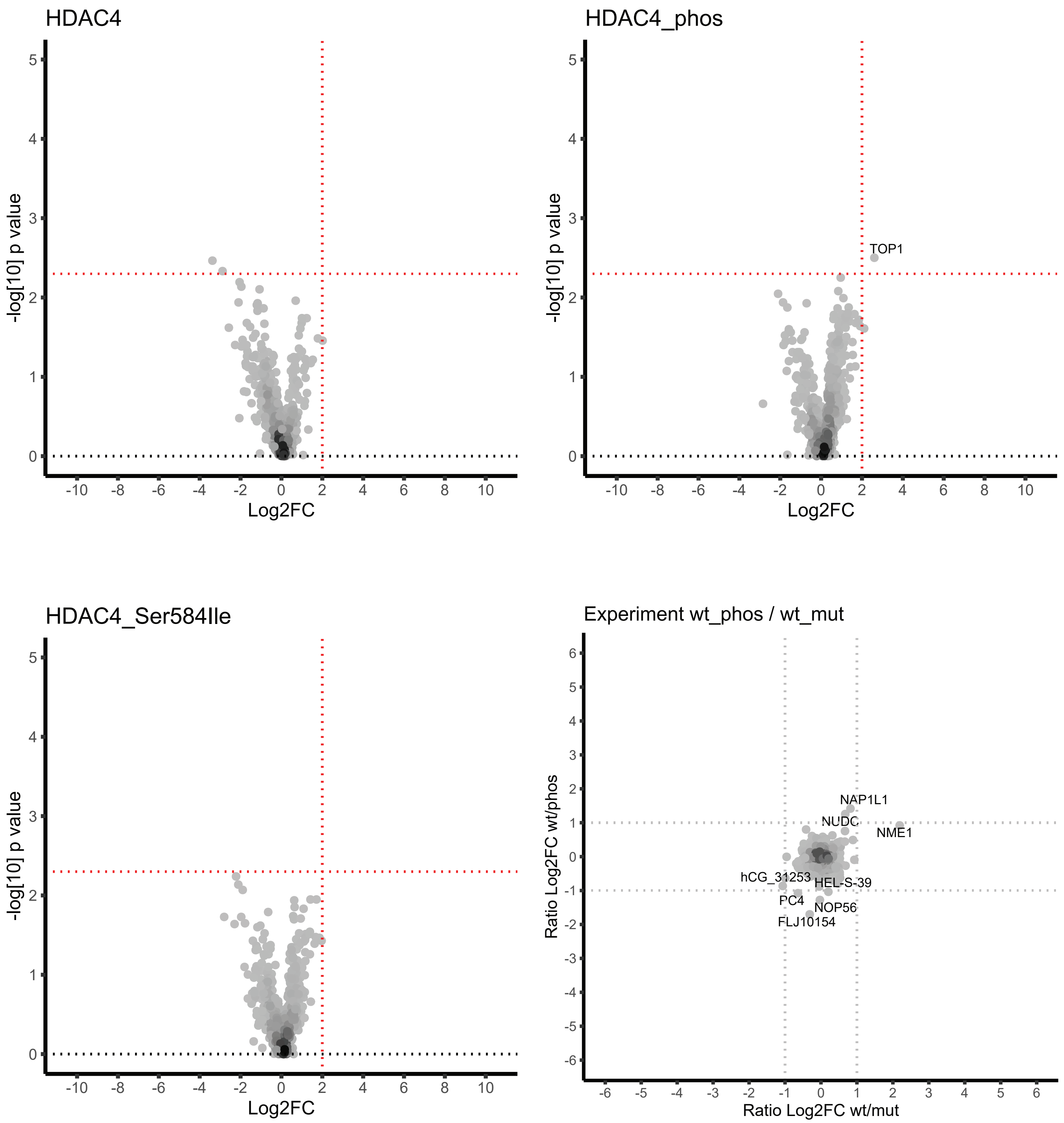

Supplementary Figure 14: Volcano and SILAC scatter plots for the HDAC4 peptide candidate: LFQ values were used to perform a Wilcoxon test where proteins identified in one peptide form (triplicates) were compared to all the proteins identified in all other WT and Mutant peptide forms. The calculated p-values and fold changes (Significance:Log2Fold change > 2 and p-value < 0.005) were plotted as Volcano plots (Top and bottom right). The log2 transformed SILAC ratios were plotted on scatter plots with wt/mut ratios on the x-axis and wt/phos ratios on the y-axis (bottom left) whereas the phos/mut ratios are not shown. SILAC ratios reveal differential interactors. To point out LFQ specific interactors in the scatter plots we used colored nodes as follows: Blue; phospho-peptide specific, yellow; wt-peptide specific, red; mut-peptide specific, orange; wt and mut-peptide specific, green; wt and phospho-peptide specific, purple; mut and phospho-peptide specific, and pink; wt, mut and phospho-peptide specific.

LRRC16A: SPKPLLQpSPKPSLAA

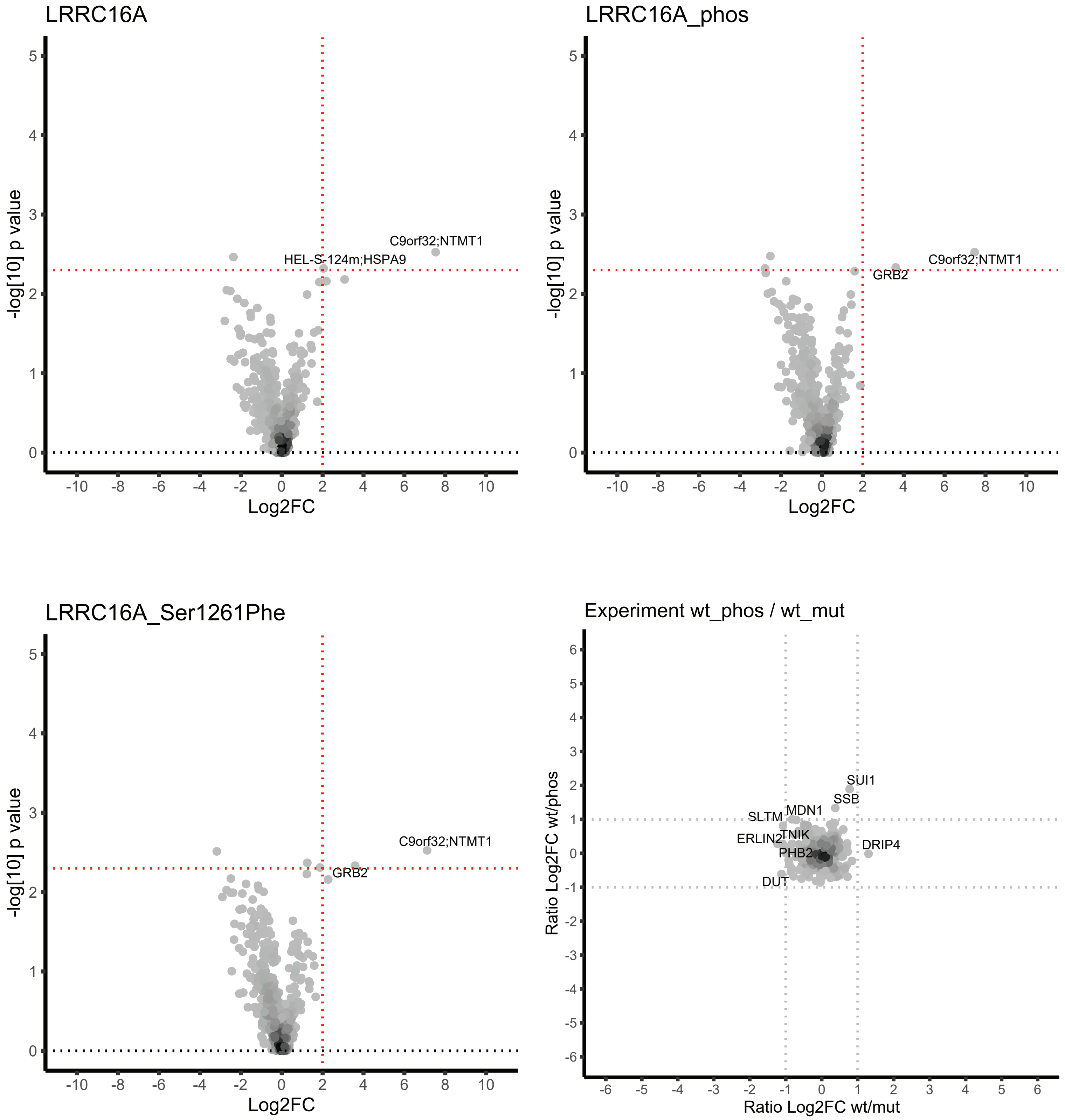

Supplementary Figure 15: Volcano and SILAC scatter plots for the LRRC16A peptide candidate: LFQ values were used to perform a Wilcoxon test where proteins identified in one peptide form (triplicates) were compared to all the proteins identified in all other WT and Mutant peptide forms. The calculated p-values and fold changes (Significance:Log2Fold change > 2 and p-value < 0.005) were plotted as Volcano plots (Top and bottom right). The log2 transformed SILAC ratios were plotted on scatter plots with wt/mut ratios on the x-axis and wt/phos ratios on the y-axis (bottom left) whereas the phos/mut ratios are not shown. SILAC ratios reveal differential interactors. To point out LFQ specific interactors in the scatter plots we used colored nodes as follows: Blue; phospho-peptide specific, yellow; wt-peptide specific, red; mut-peptide specific, orange; wt and mut-peptide specific, green; wt and phospho-peptide specific, purple; mut and phospho-peptide specific, and pink; wt, mut and phospho-peptide specific.

MAFB: PLSTPCSpSVPSSPSF

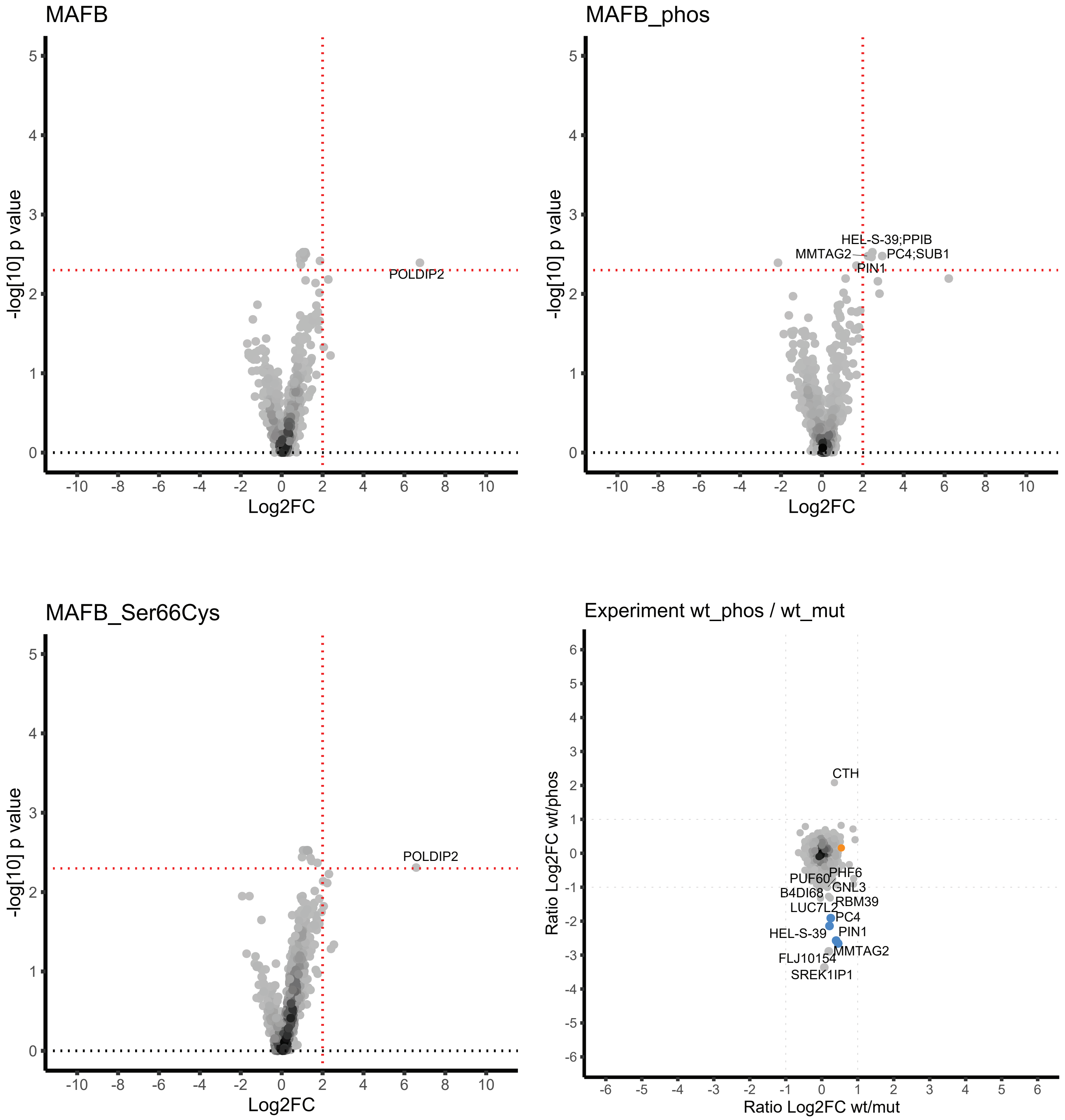

Supplementary Figure 16: Volcano and SILAC scatter plots for the MAFB_Ser66Cys peptide candidate: LFQ values were used to perform a Wilcoxon test where proteins identified in one peptide form (triplicates) were compared to all the proteins identified in all other WT and Mutant peptide forms. The calculated p-values and fold changes (Significance:Log2Fold change > 2 and p-value < 0.005) were plotted as Volcano plots (Top and bottom right). The log2 transformed SILAC ratios were plotted on scatter plots with wt/mut ratios on the x-axis and wt/phos ratios on the y-axis (bottom left) whereas the phos/mut ratios are not shown. SILAC ratios reveal differential interactors. To point out LFQ specific interactors in the scatter plots we used colored nodes as follows: Blue; phospho-peptide specific, yellow; wt-peptide specific, red; mut-peptide specific, orange; wt and mut-peptide specific, green; wt and phospho-peptide specific, purple; mut and phospho-peptide specific, and pink; wt, mut and phospho-peptide specific.

MAFB: TPCSSVPpSSPSFSPT

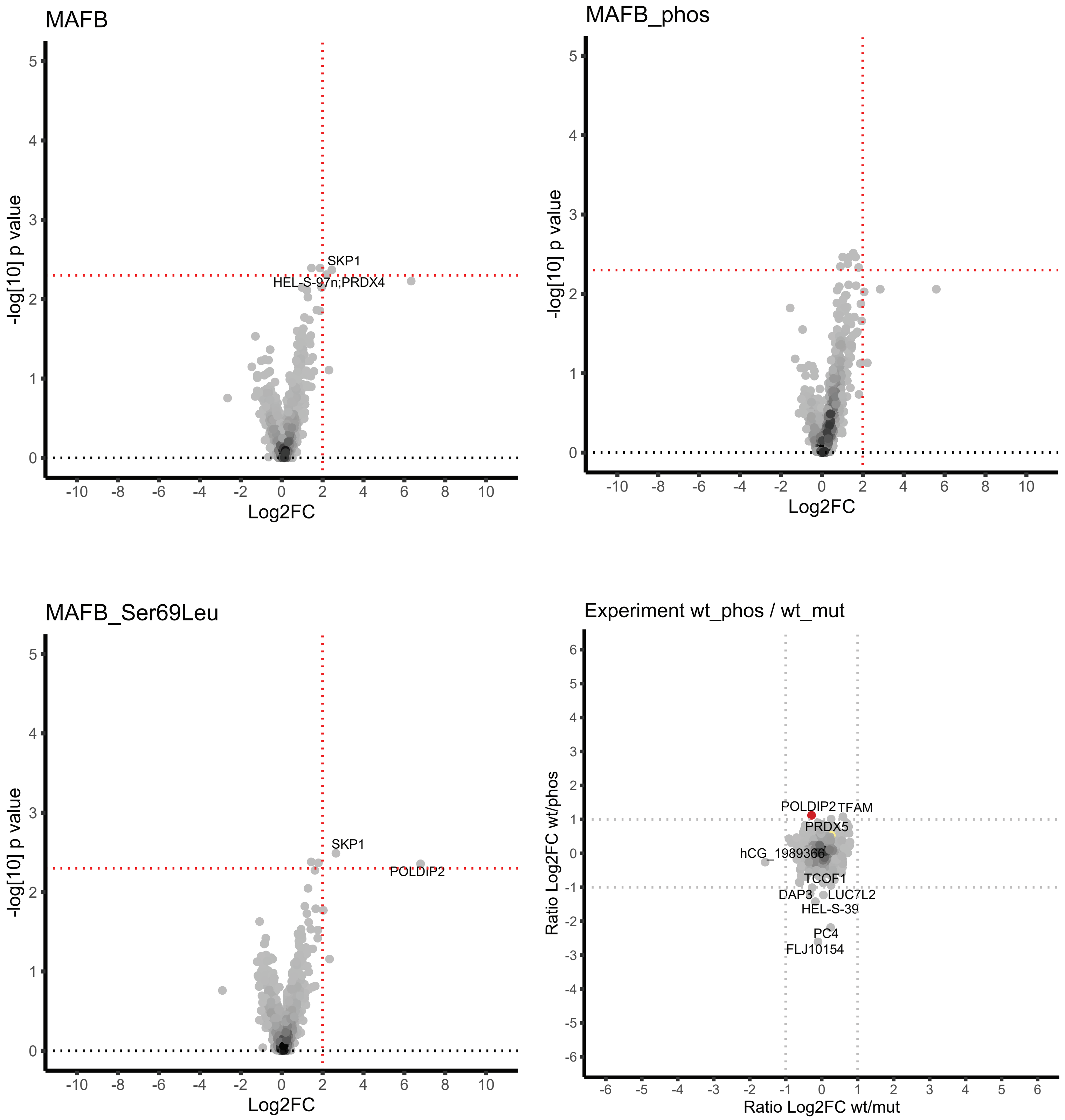

Supplementary Figure 17: Volcano and SILAC scatter plots for the MAFB_Ser69Leu peptide candidate: LFQ values were used to perform a Wilcoxon test where proteins identified in one peptide form (triplicates) were compared to all the proteins identified in all other WT and Mutant peptide forms. The calculated p-values and fold changes (Significance:Log2Fold change > 2 and p-value < 0.005) were plotted as Volcano plots (Top and bottom right). The log2 transformed SILAC ratios were plotted on scatter plots with wt/mut ratios on the x-axis and wt/phos ratios on the y-axis (bottom left) whereas the phos/mut ratios are not shown. SILAC ratios reveal differential interactors. To point out LFQ specific interactors in the scatter plots we used colored nodes as follows: Blue; phospho-peptide specific, yellow; wt-peptide specific, red; mut-peptide specific, orange; wt and mut-peptide specific, green; wt and phospho-peptide specific, purple; mut and phospho-peptide specific, and pink; wt, mut and phospho-peptide specific.

MAFB: PCSSVPSpSPSFSPTE

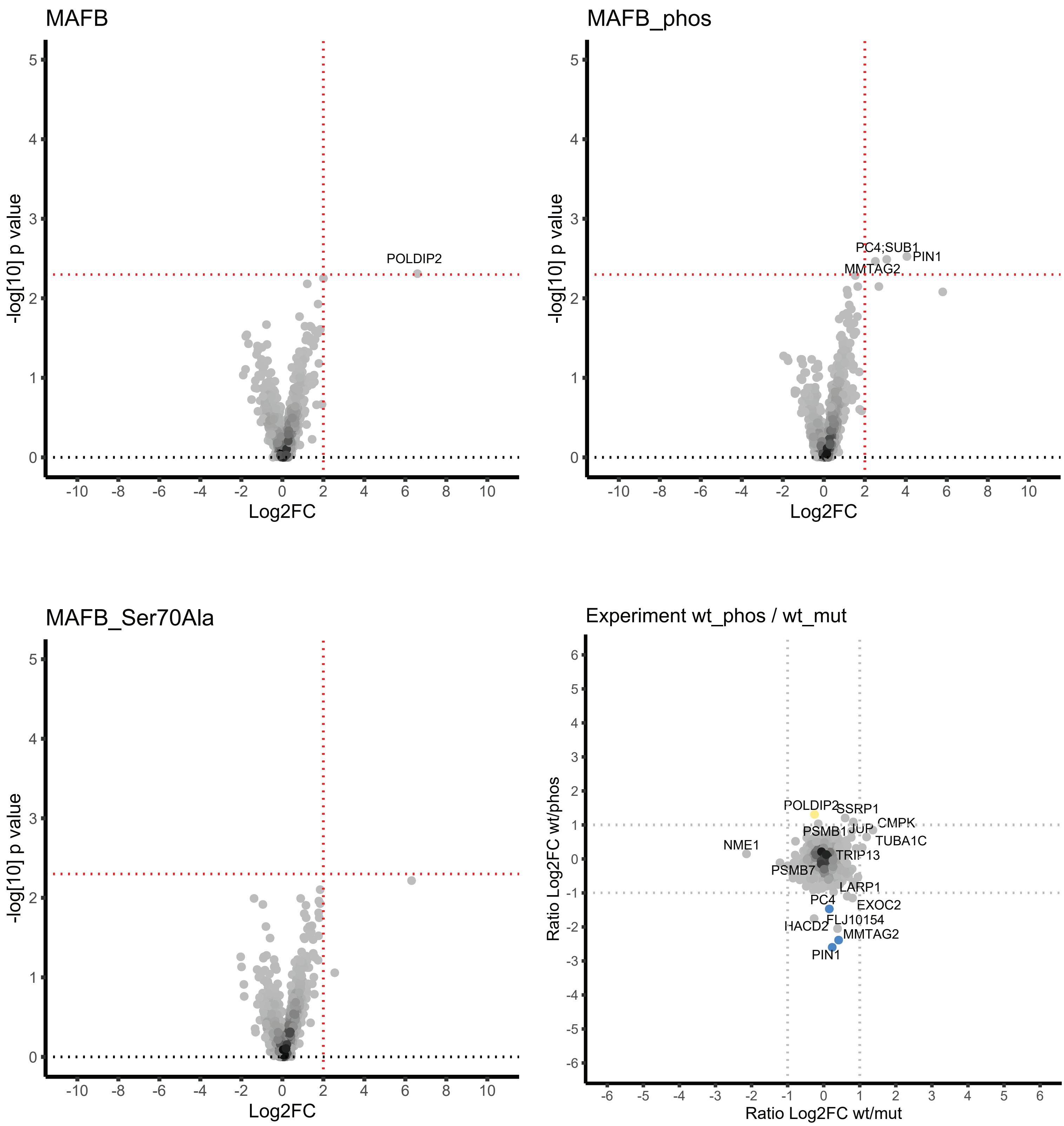

Supplementary Figure 18: Volcano and SILAC scatter plots for the MAFB_Ser70Ala peptide candidate: LFQ values were used to perform a Wilcoxon test where proteins identified in one peptide form (triplicates) were compared to all the proteins identified in all other WT and Mutant peptide forms. The calculated p-values and fold changes (Significance:Log2Fold change > 2 and p-value < 0.005) were plotted as Volcano plots (Top and bottom right). The log2 transformed SILAC ratios were plotted on scatter plots with wt/mut ratios on the x-axis and wt/phos ratios on the y-axis (bottom left) whereas the phos/mut ratios are not shown. SILAC ratios reveal differential interactors. To point out LFQ specific interactors in the scatter plots we used colored nodes as follows: Blue; phospho-peptide specific, yellow; wt-peptide specific, red; mut-peptide specific, orange; wt and mut-peptide specific, green; wt and phospho-peptide specific, purple; mut and phospho-peptide specific, and pink; wt, mut and phospho-peptide specific.

MAFB: VSSTPLSpTPCSSVPS

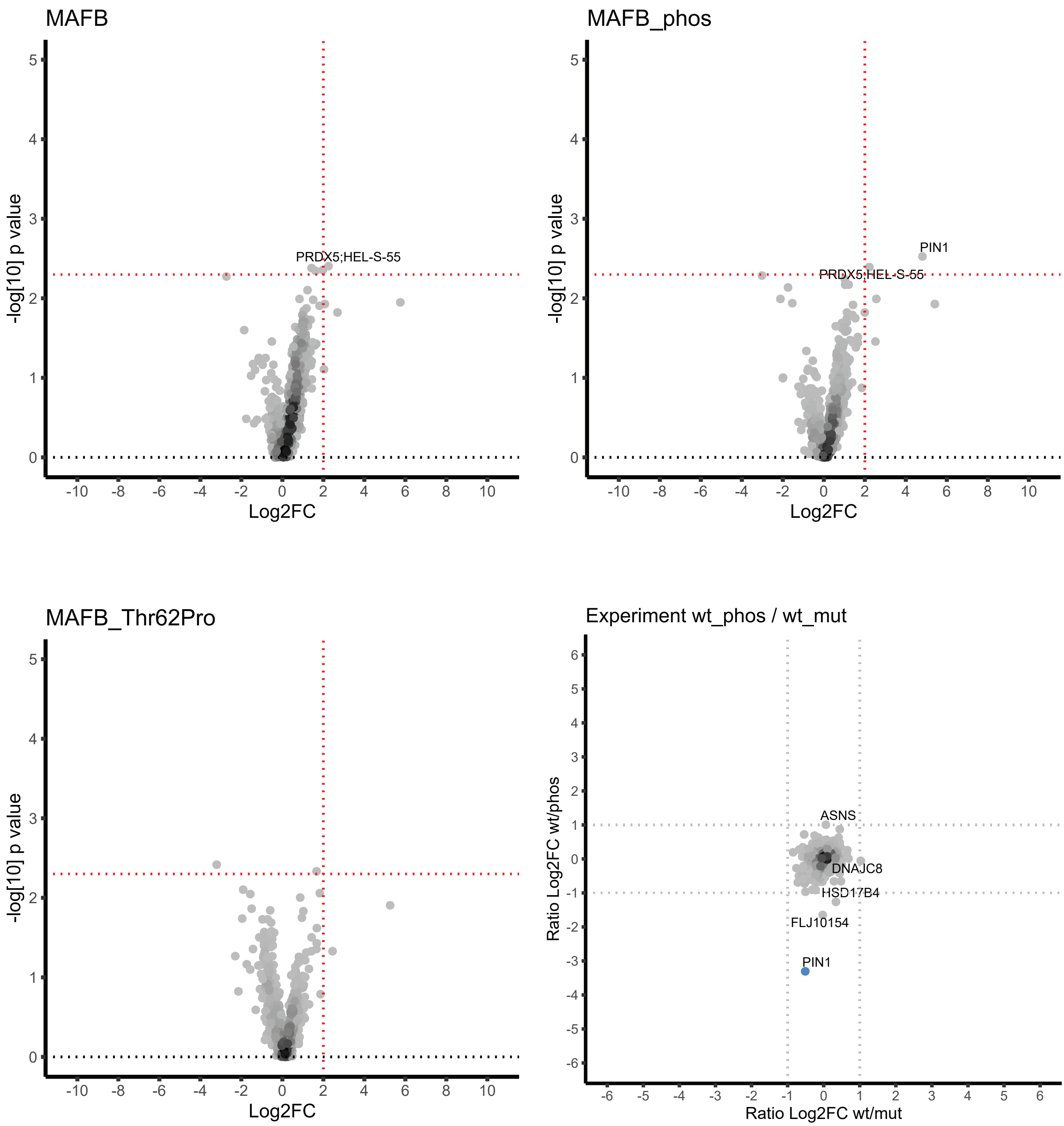

Supplementary Figure 19: Volcano and SILAC scatter plots for the MAFB_Thr62Pro peptide candidate: LFQ values were used to perform a Wilcoxon test where proteins identified in one peptide form (triplicates) were compared to all the proteins identified in all other WT and Mutant peptide forms. The calculated p-values and fold changes (Significance:Log2Fold change > 2 and p-value < 0.005) were plotted as Volcano plots (Top and bottom right). The log2 transformed SILAC ratios were plotted on scatter plots with wt/mut ratios on the x-axis and wt/phos ratios on the y-axis (bottom left) whereas the phos/mut ratios are not shown. SILAC ratios reveal differential interactors. To point out LFQ specific interactors in the scatter plots we used colored nodes as follows: Blue; phospho-peptide specific, yellow; wt-peptide specific, red; mut-peptide specific, orange; wt and mut-peptide specific, green; wt and phospho-peptide specific, purple; mut and phospho-peptide specific, and pink; wt, mut and phospho-peptide specific.

MLH1: SGSSDKVpYAHQMVRT

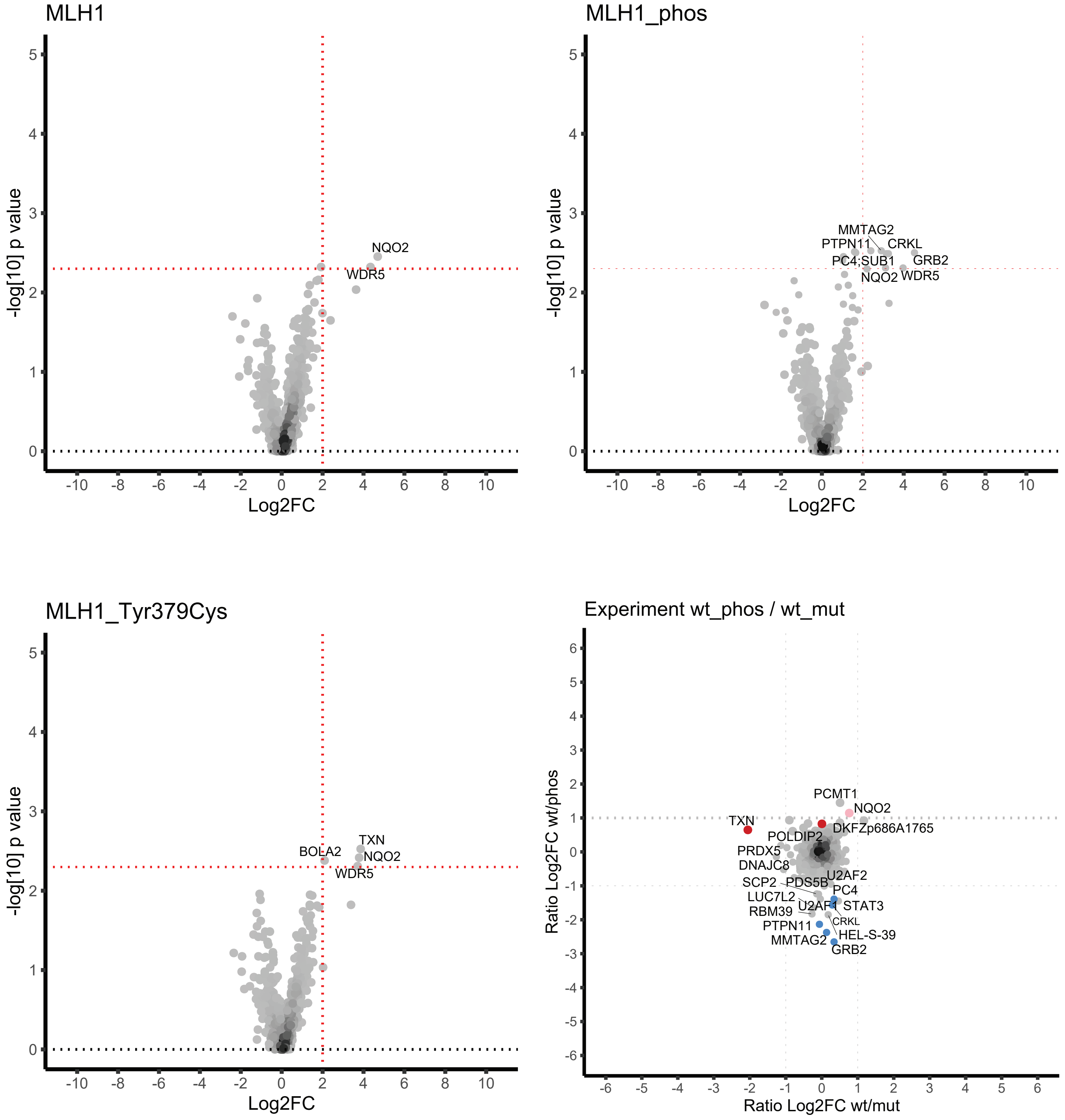

Supplementary Figure 20: Volcano and SILAC scatter plots for the MLH1 peptide candidate: LFQ values were used to perform a Wilcoxon test where proteins identified in one peptide form (triplicates) were compared to all the proteins identified in all other WT and Mutant peptide forms. The calculated p-values and fold changes (Significance:Log2Fold change > 2 and p-value < 0.005) were plotted as Volcano plots (Top and bottom right). The log2 transformed SILAC ratios were plotted on scatter plots with wt/mut ratios on the x-axis and wt/phos ratios on the y-axis (bottom left) whereas the phos/mut ratios are not shown. SILAC ratios reveal differential interactors. To point out LFQ specific interactors in the scatter plots we used colored nodes as follows: Blue; phospho-peptide specific, yellow; wt-peptide specific, red; mut-peptide specific, orange; wt and mut-peptide specific, green; wt and phospho-peptide specific, purple; mut and phospho-peptide specific, and pink; wt, mut and phospho-peptide specific.

MMAA: LSRDMNApYIRPSPTR

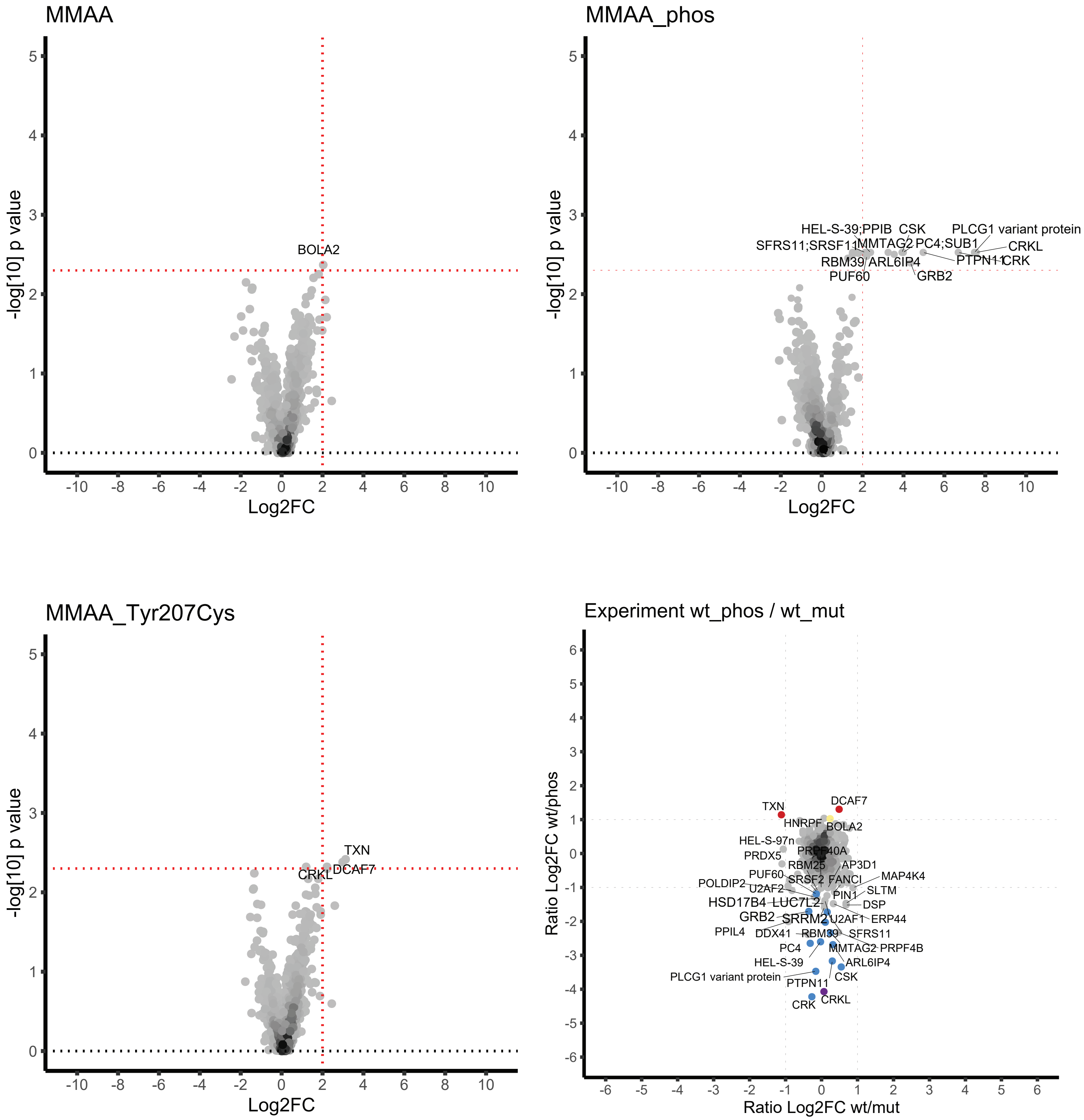

Supplementary Figure 21: Volcano and SILAC scatter plots for the MMAA peptide candidate: LFQ values were used to perform a Wilcoxon test where proteins identified in one peptide form (triplicates) were compared to all the proteins identified in all other WT and Mutant peptide forms. The calculated p-values and fold changes (Significance:Log2Fold change > 2 and p-value < 0.005) were plotted as Volcano plots (Top and bottom right). The log2 transformed SILAC ratios were plotted on scatter plots with wt/mut ratios on the x-axis and wt/phos ratios on the y-axis (bottom left) whereas the phos/mut ratios are not shown. SILAC ratios reveal differential interactors. To point out LFQ specific interactors in the scatter plots we used colored nodes as follows: Blue; phospho-peptide specific, yellow; wt-peptide specific, red; mut-peptide specific, orange; wt and mut-peptide specific, green; wt and phospho-peptide specific, purple; mut and phospho-peptide specific, and pink; wt, mut and phospho-peptide specific.

MYBPC3: GPGAPVTpTTEPVTVQ

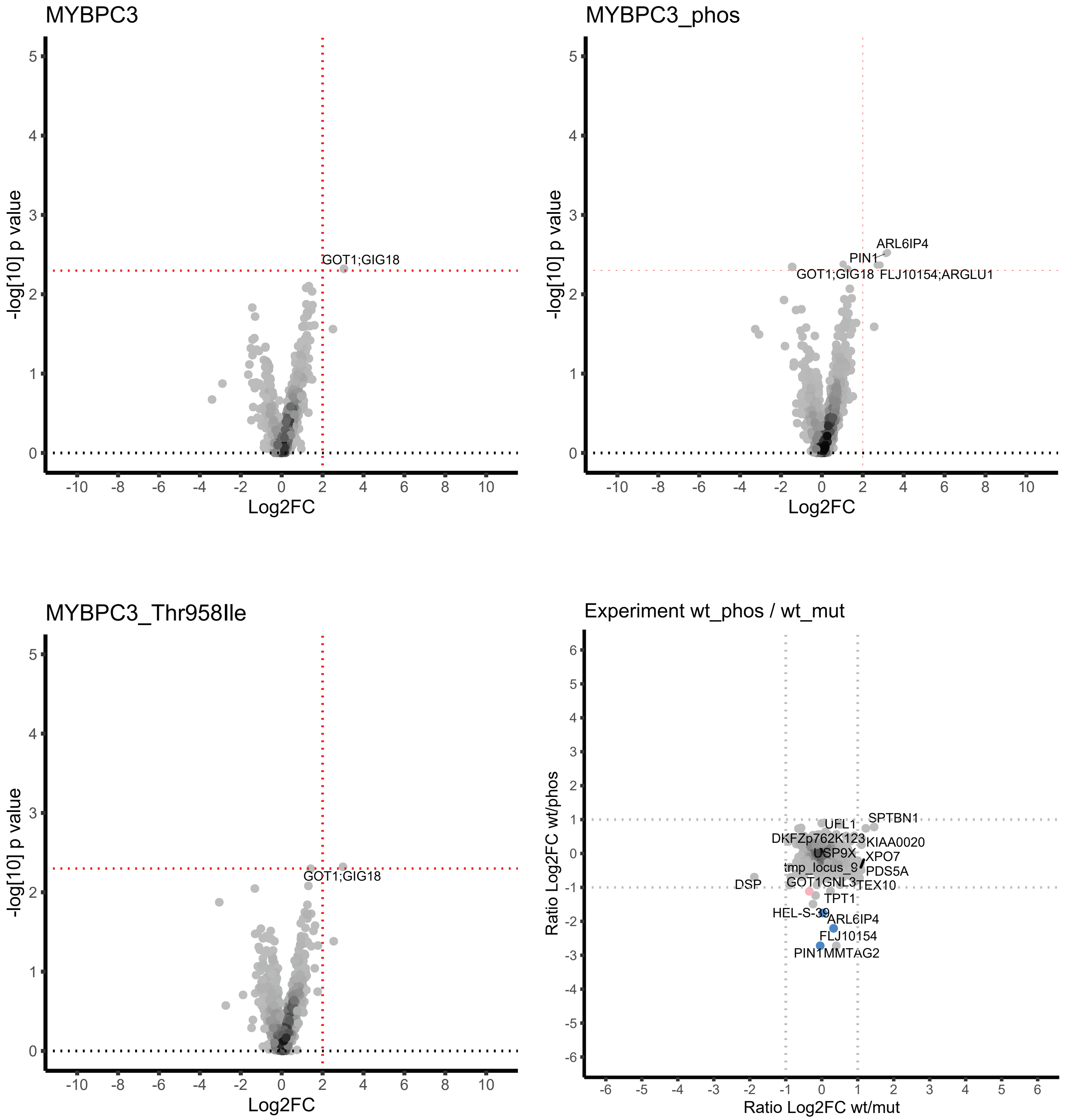

Supplementary Figure 22: Volcano and SILAC scatter plots for the MYBPC3 peptide candidate: LFQ values were used to perform a Wilcoxon test where proteins identified in one peptide form (triplicates) were compared to all the proteins identified in all other WT and Mutant peptide forms. The calculated p-values and fold changes (Significance:Log2Fold change > 2 and p-value < 0.005) were plotted as Volcano plots (Top and bottom right). The log2 transformed SILAC ratios were plotted on scatter plots with wt/mut ratios on the x-axis and wt/phos ratios on the y-axis (bottom left) whereas the phos/mut ratios are not shown. SILAC ratios reveal differential interactors. To point out LFQ specific interactors in the scatter plots we used colored nodes as follows: Blue; phospho-peptide specific, yellow; wt-peptide specific, red; mut-peptide specific, orange; wt and mut-peptide specific, green; wt and phospho-peptide specific, purple; mut and phospho-peptide specific, and pink; wt, mut and phospho-peptide specific.

MYT1L: SNSLEDDpSDKNENLG

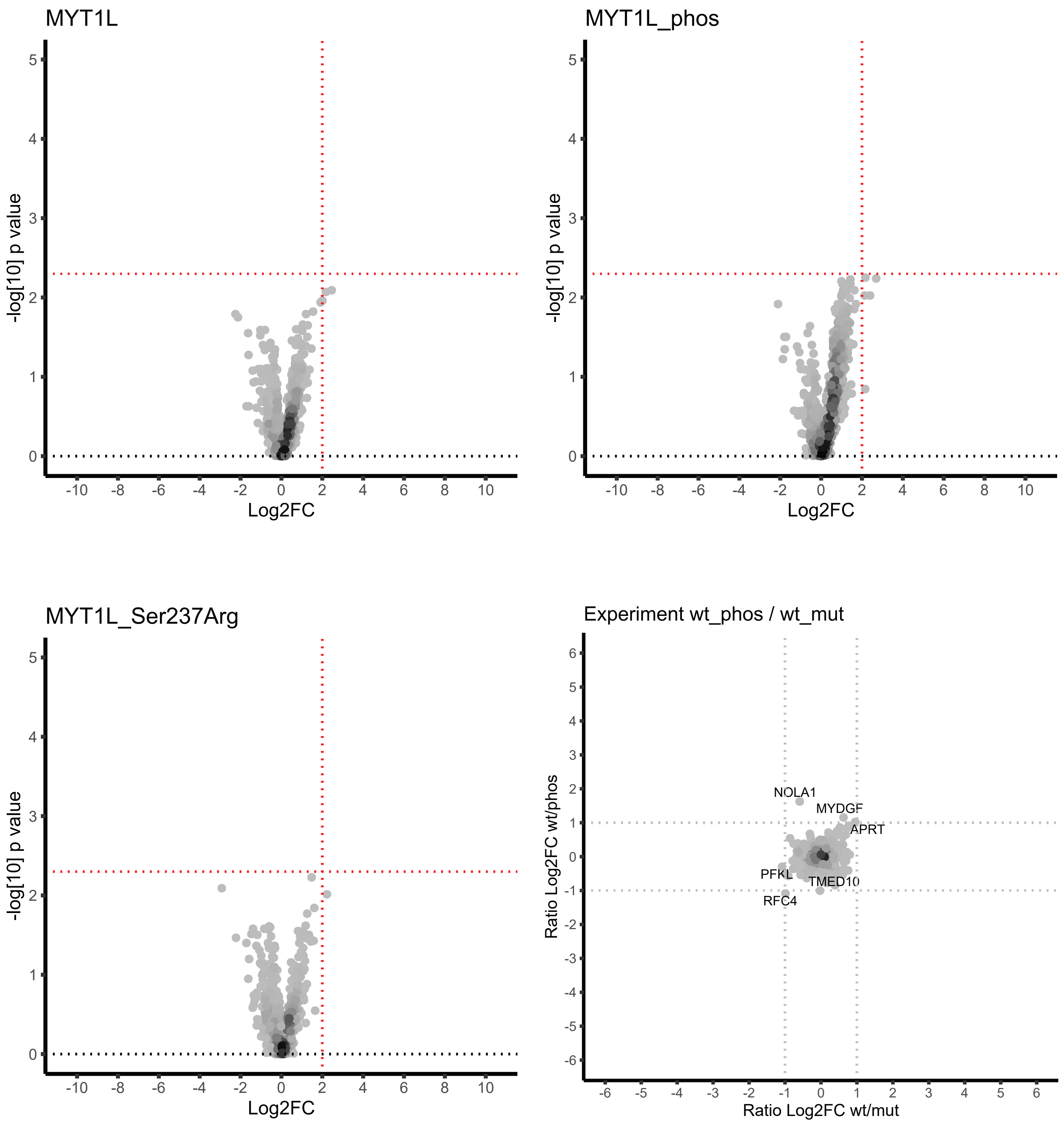

Supplementary Figure 23: Volcano and SILAC scatter plots for the MYT1L peptide candidate: LFQ values were used to perform a Wilcoxon test where proteins identified in one peptide form (triplicates) were compared to all the proteins identified in all other WT and Mutant peptide forms. The calculated p-values and fold changes (Significance:Log2Fold change > 2 and p-value < 0.005) were plotted as Volcano plots (Top and bottom right). The log2 transformed SILAC ratios were plotted on scatter plots with wt/mut ratios on the x-axis and wt/phos ratios on the y-axis (bottom left) whereas the phos/mut ratios are not shown. SILAC ratios reveal differential interactors. To point out LFQ specific interactors in the scatter plots we used colored nodes as follows: Blue; phospho-peptide specific, yellow; wt-peptide specific, red; mut-peptide specific, orange; wt and mut-peptide specific, green; wt and phospho-peptide specific, purple; mut and phospho-peptide specific, and pink; wt, mut and phospho-peptide specific.

NCOA6: PSPGRQNpSKAPKLTL

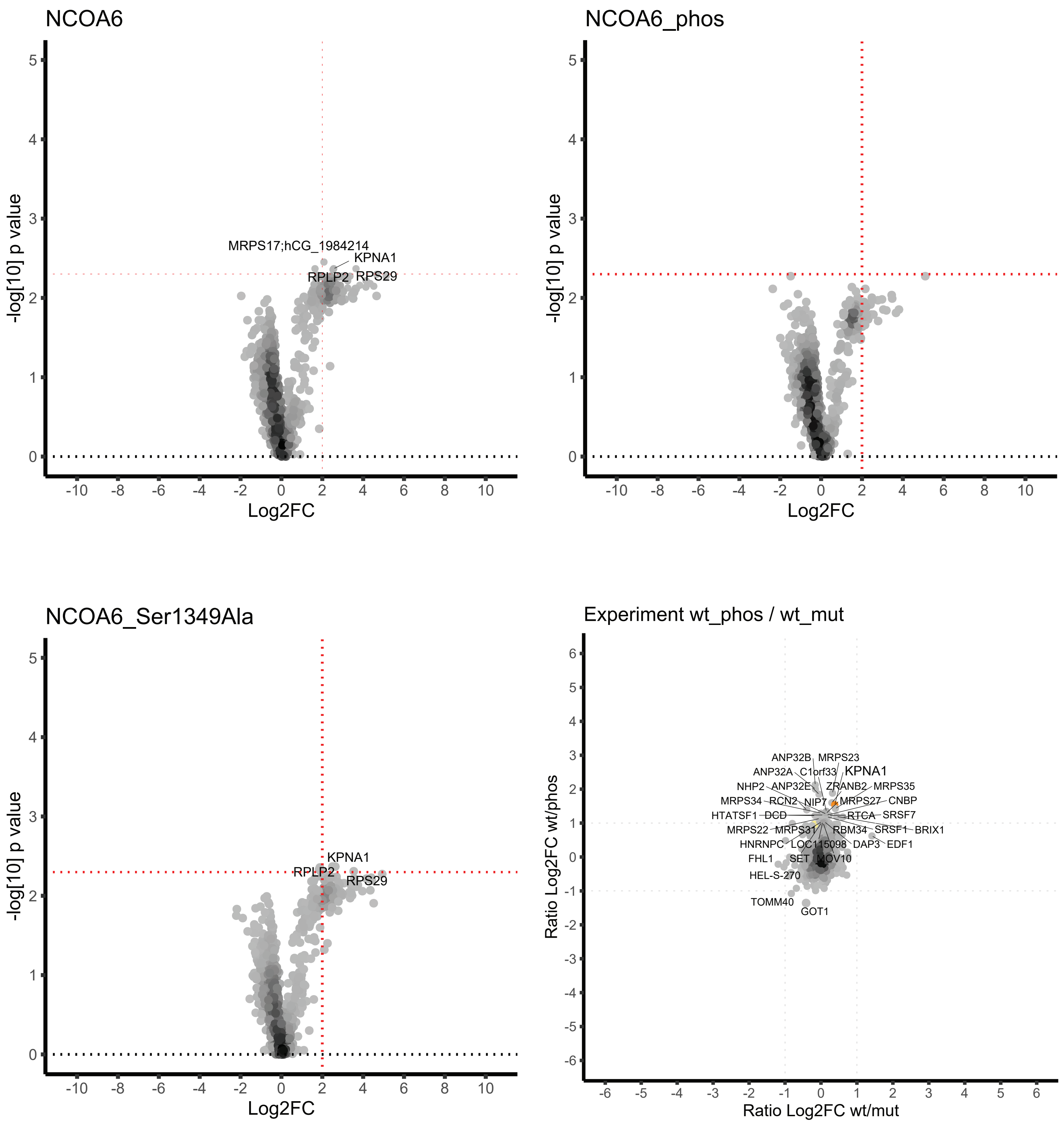

Supplementary Figure 24: Volcano and SILAC scatter plots for the NCOA6 peptide candidate: LFQ values were used to perform a Wilcoxon test where proteins identified in one peptide form (triplicates) were compared to all the proteins identified in all other WT and Mutant peptide forms. The calculated p-values and fold changes (Significance:Log2Fold change > 2 and p-value < 0.005) were plotted as Volcano plots (Top and bottom right). The log2 transformed SILAC ratios were plotted on scatter plots with wt/mut ratios on the x-axis and wt/phos ratios on the y-axis (bottom left) whereas the phos/mut ratios are not shown. SILAC ratios reveal differential interactors. To point out LFQ specific interactors in the scatter plots we used colored nodes as follows: Blue; phospho-peptide specific, yellow; wt-peptide specific, red; mut-peptide specific, orange; wt and mut-peptide specific, green; wt and phospho-peptide specific, purple; mut and phospho-peptide specific, and pink; wt, mut and phospho-peptide specific.

NRAP: EQPRGKGpSFPAMITP

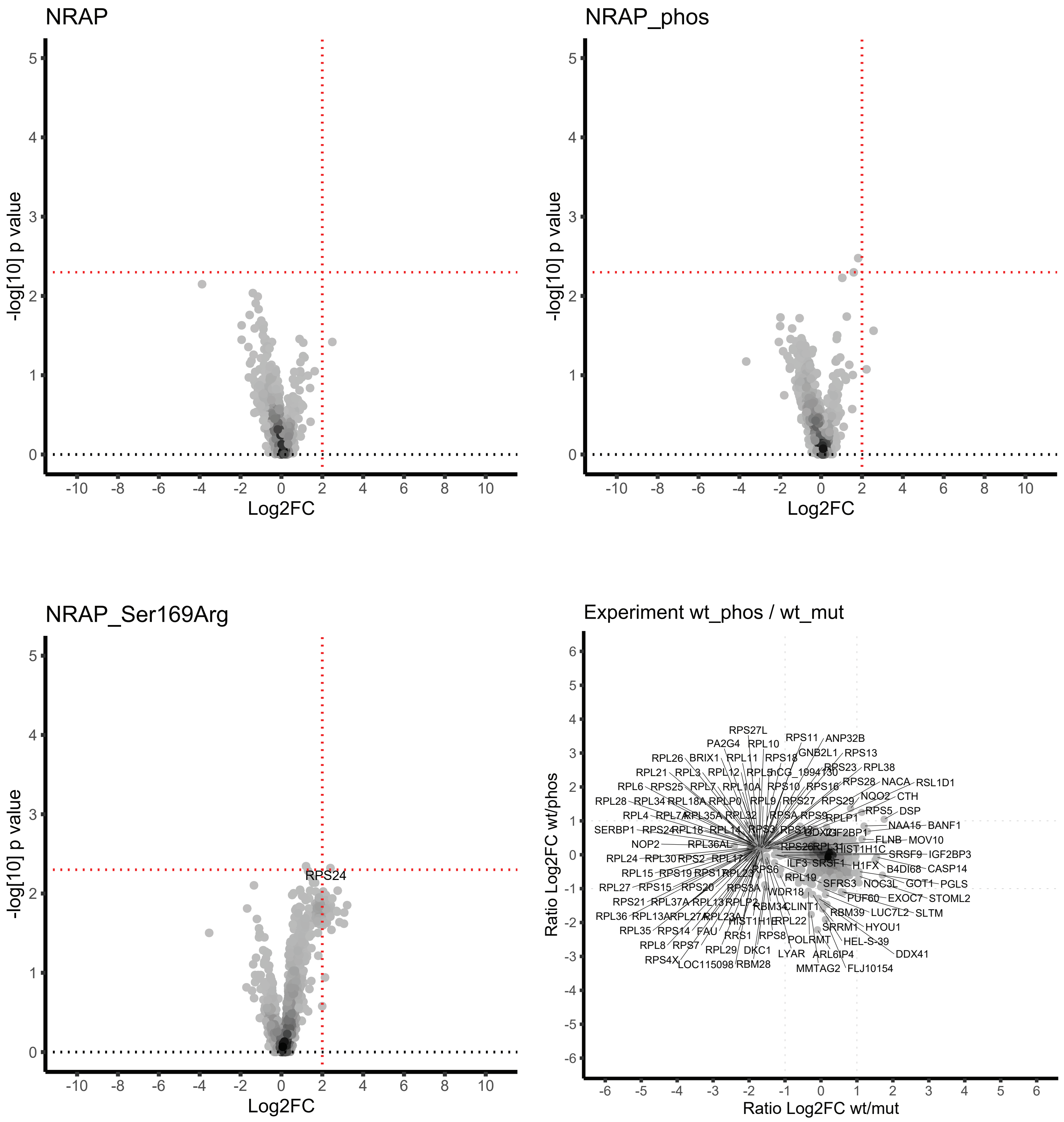

Supplementary Figure 25: Volcano and SILAC scatter plots for the NRAP peptide candidate: LFQ values were used to perform a Wilcoxon test where proteins identified in one peptide form (triplicates) were compared to all the proteins identified in all other WT and Mutant peptide forms. The calculated p-values and fold changes (Significance:Log2Fold change > 2 and p-value < 0.005) were plotted as Volcano plots (Top and bottom right). The log2 transformed SILAC ratios were plotted on scatter plots with wt/mut ratios on the x-axis and wt/phos ratios on the y-axis (bottom left) whereas the phos/mut ratios are not shown. SILAC ratios reveal differential interactors. To point out LFQ specific interactors in the scatter plots we used colored nodes as follows: Blue; phospho-peptide specific, yellow; wt-peptide specific, red; mut-peptide specific, orange; wt and mut-peptide specific, green; wt and phospho-peptide specific, purple; mut and phospho-peptide specific, and pink; wt, mut and phospho-peptide specific.

NRCAM: PSEASEQpYLTKASEP

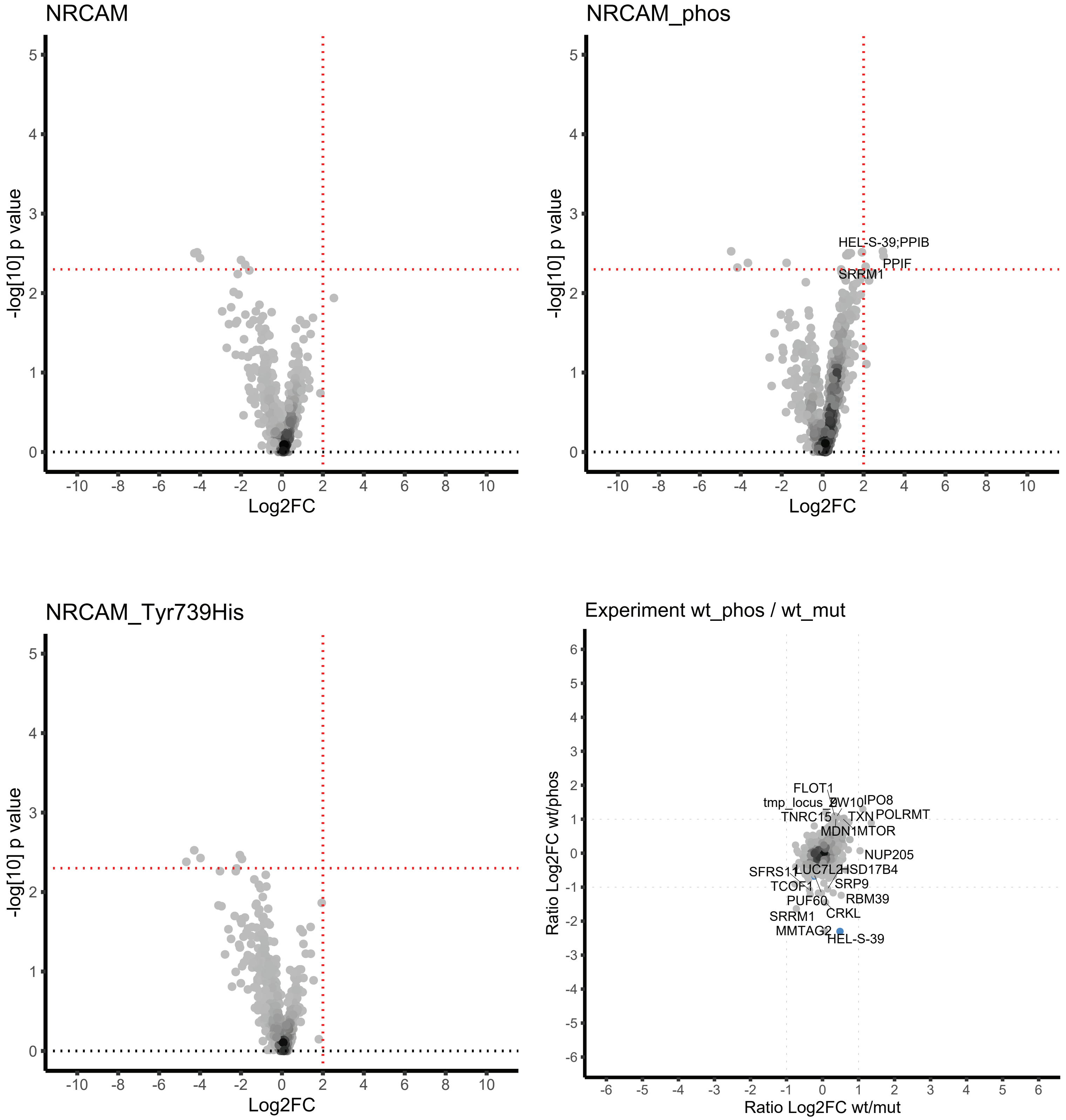

Supplementary Figure 26: Volcano and SILAC scatter plots for the NRCAM peptide candidate: LFQ values were used to perform a Wilcoxon test where proteins identified in one peptide form (triplicates) were compared to all the proteins identified in all other WT and Mutant peptide forms. The calculated p-values and fold changes (Significance:Log2Fold change > 2 and p-value < 0.005) were plotted as Volcano plots (Top and bottom right). The log2 transformed SILAC ratios were plotted on scatter plots with wt/mut ratios on the x-axis and wt/phos ratios on the y-axis (bottom left) whereas the phos/mut ratios are not shown. SILAC ratios reveal differential interactors. To point out LFQ specific interactors in the scatter plots we used colored nodes as follows: Blue; phospho-peptide specific, yellow; wt-peptide specific, red; mut-peptide specific, orange; wt and mut-peptide specific, green; wt and phospho-peptide specific, purple; mut and phospho-peptide specific, and pink; wt, mut and phospho-peptide specific.

PHF20: PENDIVKpSPQENLRE

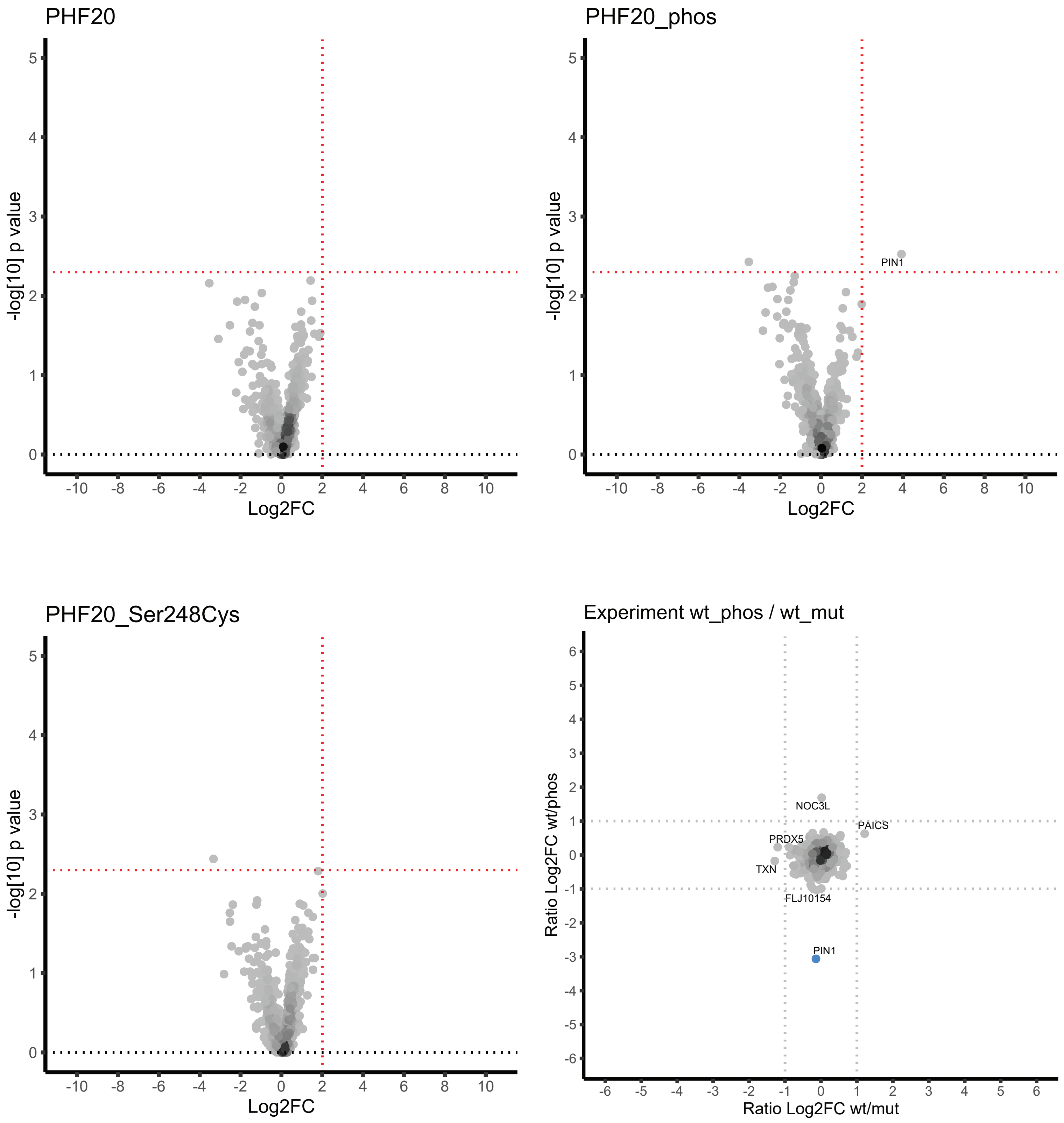

Supplementary Figure 27: Volcano and SILAC scatter plots for the PHF20 peptide candidate: LFQ values were used to perform a Wilcoxon test where proteins identified in one peptide form (triplicates) were compared to all the proteins identified in all other WT and Mutant peptide forms. The calculated p-values and fold changes (Significance:Log2Fold change > 2 and p-value < 0.005) were plotted as Volcano plots (Top and bottom right). The log2 transformed SILAC ratios were plotted on scatter plots with wt/mut ratios on the x-axis and wt/phos ratios on the y-axis (bottom left) whereas the phos/mut ratios are not shown. SILAC ratios reveal differential interactors. To point out LFQ specific interactors in the scatter plots we used colored nodes as follows: Blue; phospho-peptide specific, yellow; wt-peptide specific, red; mut-peptide specific, orange; wt and mut-peptide specific, green; wt and phospho-peptide specific, purple; mut and phospho-peptide specific, and pink; wt, mut and phospho-peptide specific.

PITPNM1: SPVEAEGpTPEPGAEA

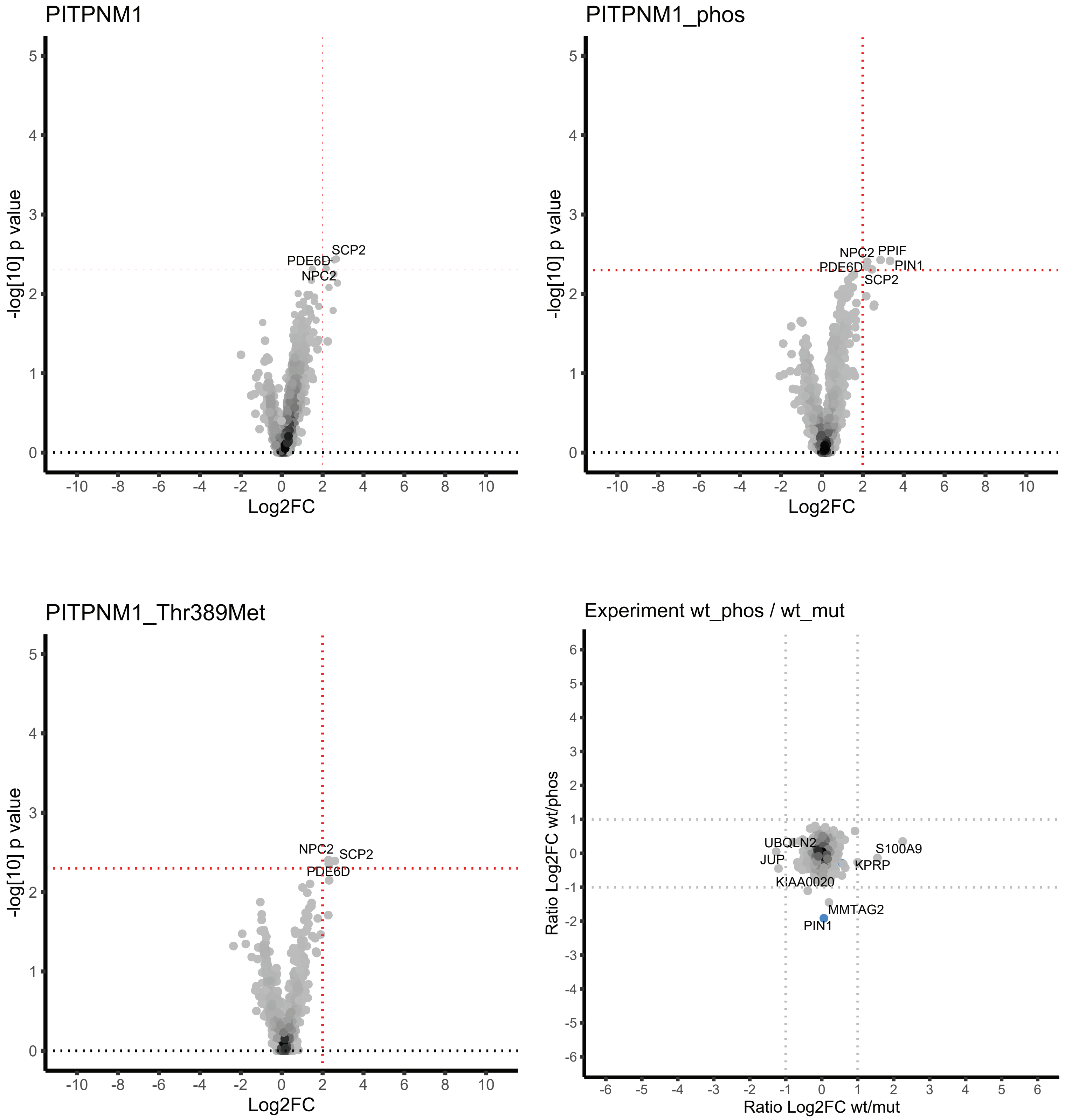

Supplementary Figure 28: Volcano and SILAC scatter plots for the PITPNM1 peptide candidate: LFQ values were used to perform a Wilcoxon test where proteins identified in one peptide form (triplicates) were compared to all the proteins identified in all other WT and Mutant peptide forms. The calculated p-values and fold changes (Significance:Log2Fold change > 2 and p-value < 0.005) were plotted as Volcano plots (Top and bottom right). The log2 transformed SILAC ratios were plotted on scatter plots with wt/mut ratios on the x-axis and wt/phos ratios on the y-axis (bottom left) whereas the phos/mut ratios are not shown. SILAC ratios reveal differential interactors. To point out LFQ specific interactors in the scatter plots we used colored nodes as follows: Blue; phospho-peptide specific, yellow; wt-peptide specific, red; mut-peptide specific, orange; wt and mut-peptide specific, green; wt and phospho-peptide specific, purple; mut and phospho-peptide specific, and pink; wt, mut and phospho-peptide specific.

PPIG: SKQLESKpSNEHDHSK

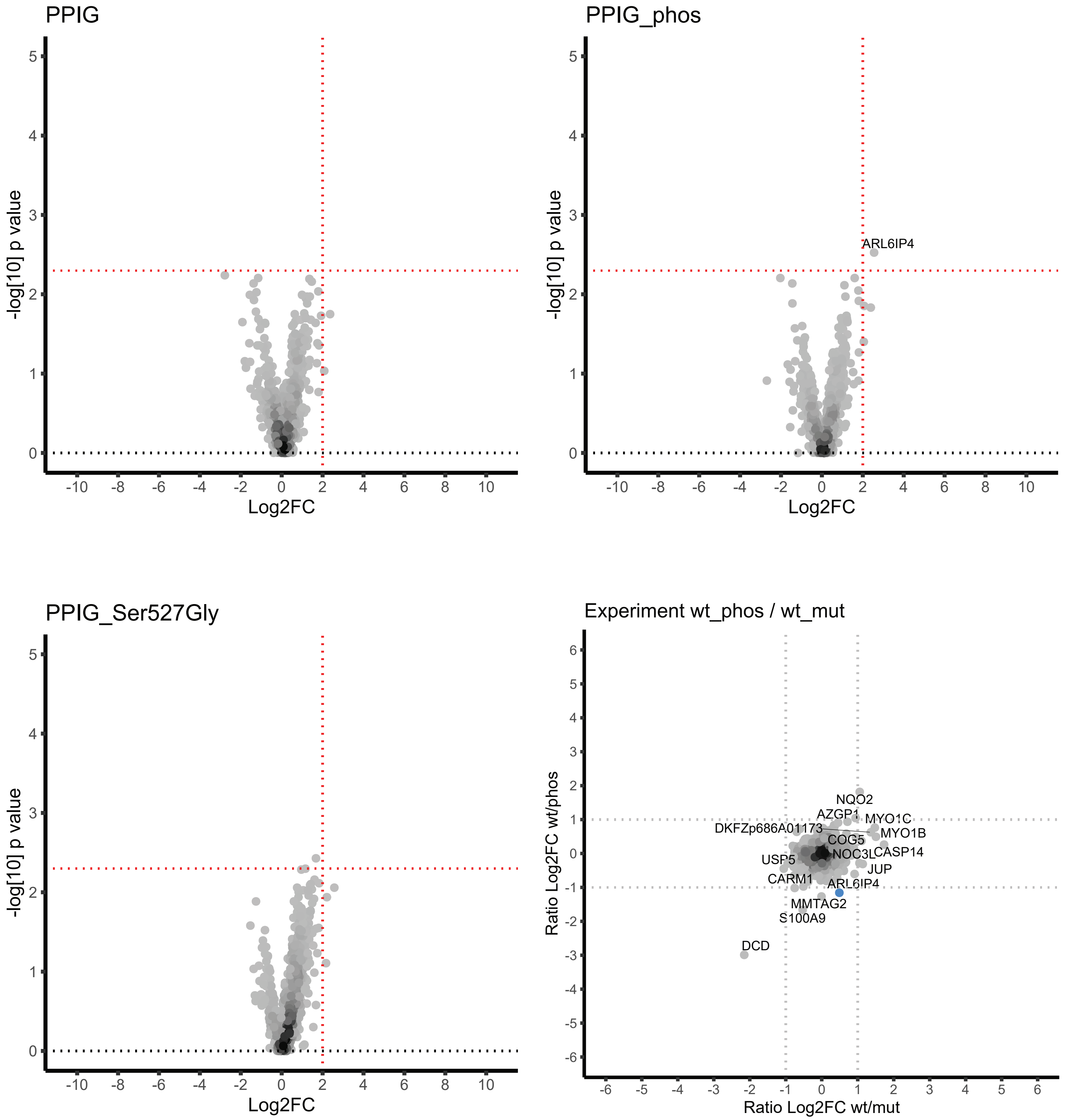

Supplementary Figure 29: Volcano and SILAC scatter plots for the PPIG peptide candidate: LFQ values were used to perform a Wilcoxon test where proteins identified in one peptide form (triplicates) were compared to all the proteins identified in all other WT and Mutant peptide forms. The calculated p-values and fold changes (Significance:Log2Fold change > 2 and p-value < 0.005) were plotted as Volcano plots (Top and bottom right). The log2 transformed SILAC ratios were plotted on scatter plots with wt/mut ratios on the x-axis and wt/phos ratios on the y-axis (bottom left) whereas the phos/mut ratios are not shown. SILAC ratios reveal differential interactors. To point out LFQ specific interactors in the scatter plots we used colored nodes as follows: Blue; phospho-peptide specific, yellow; wt-peptide specific, red; mut-peptide specific, orange; wt and mut-peptide specific, green; wt and phospho-peptide specific, purple; mut and phospho-peptide specific, and pink; wt, mut and phospho-peptide specific.

SCN1A: DEFQKSEpSEDSIRRK

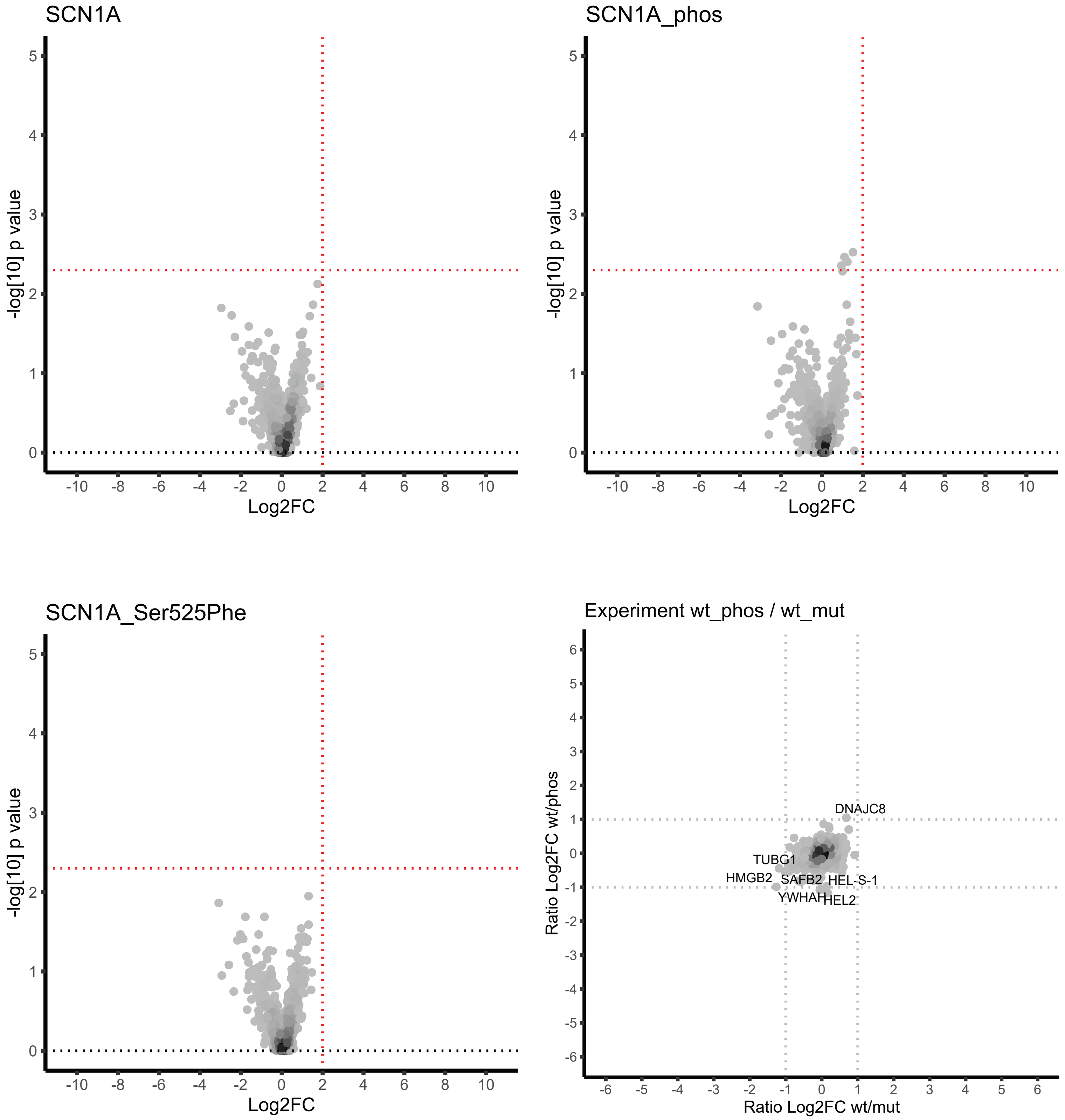

Supplementary Figure 30: Volcano and SILAC scatter plots for the SCN1A peptide candidate: LFQ values were used to perform a Wilcoxon test where proteins identified in one peptide form (triplicates) were compared to all the proteins identified in all other WT and Mutant peptide forms. The calculated p-values and fold changes (Significance:Log2Fold change > 2 and p-value < 0.005) were plotted as Volcano plots (Top and bottom right). The log2 transformed SILAC ratios were plotted on scatter plots with wt/mut ratios on the x-axis and wt/phos ratios on the y-axis (bottom left) whereas the phos/mut ratios are not shown. SILAC ratios reveal differential interactors. To point out LFQ specific interactors in the scatter plots we used colored nodes as follows: Blue; phospho-peptide specific, yellow; wt-peptide specific, red; mut-peptide specific, orange; wt and mut-peptide specific, green; wt and phospho-peptide specific, purple; mut and phospho-peptide specific, and pink; wt, mut and phospho-peptide specific.

SCN2A: GQLLPEGpTTTETEIR

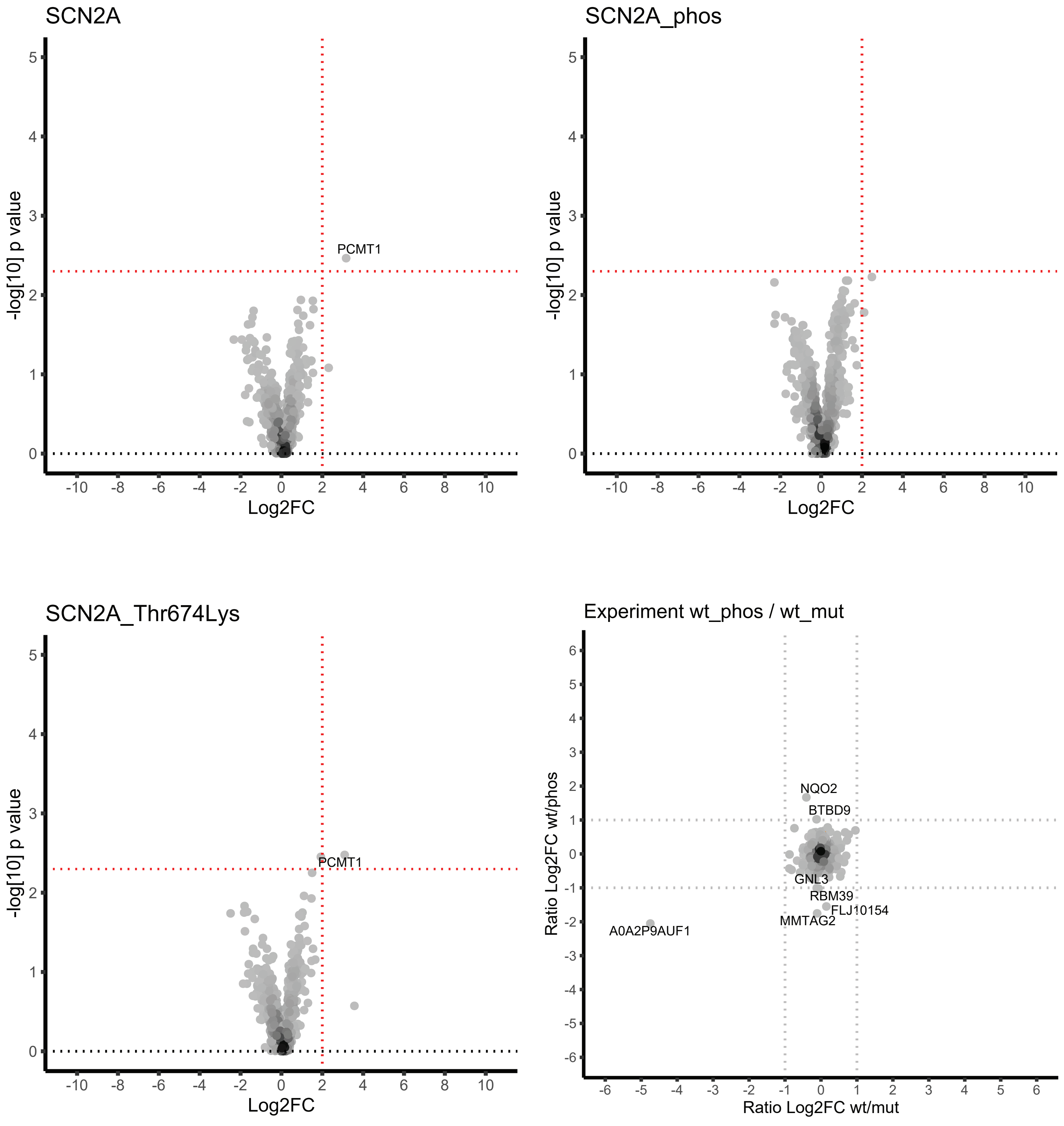

Supplementary Figure 31: Volcano and SILAC scatter plots for the SCN2A peptide candidate: LFQ values were used to perform a Wilcoxon test where proteins identified in one peptide form (triplicates) were compared to all the proteins identified in all other WT and Mutant peptide forms. The calculated p-values and fold changes (Significance:Log2Fold change > 2 and p-value < 0.005) were plotted as Volcano plots (Top and bottom right). The log2 transformed SILAC ratios were plotted on scatter plots with wt/mut ratios on the x-axis and wt/phos ratios on the y-axis (bottom left) whereas the phos/mut ratios are not shown. SILAC ratios reveal differential interactors. To point out LFQ specific interactors in the scatter plots we used colored nodes as follows: Blue; phospho-peptide specific, yellow; wt-peptide specific, red; mut-peptide specific, orange; wt and mut-peptide specific, green; wt and phospho-peptide specific, purple; mut and phospho-peptide specific, and pink; wt, mut and phospho-peptide specific.

SPRY4: GAPELAPpTPARCDQD

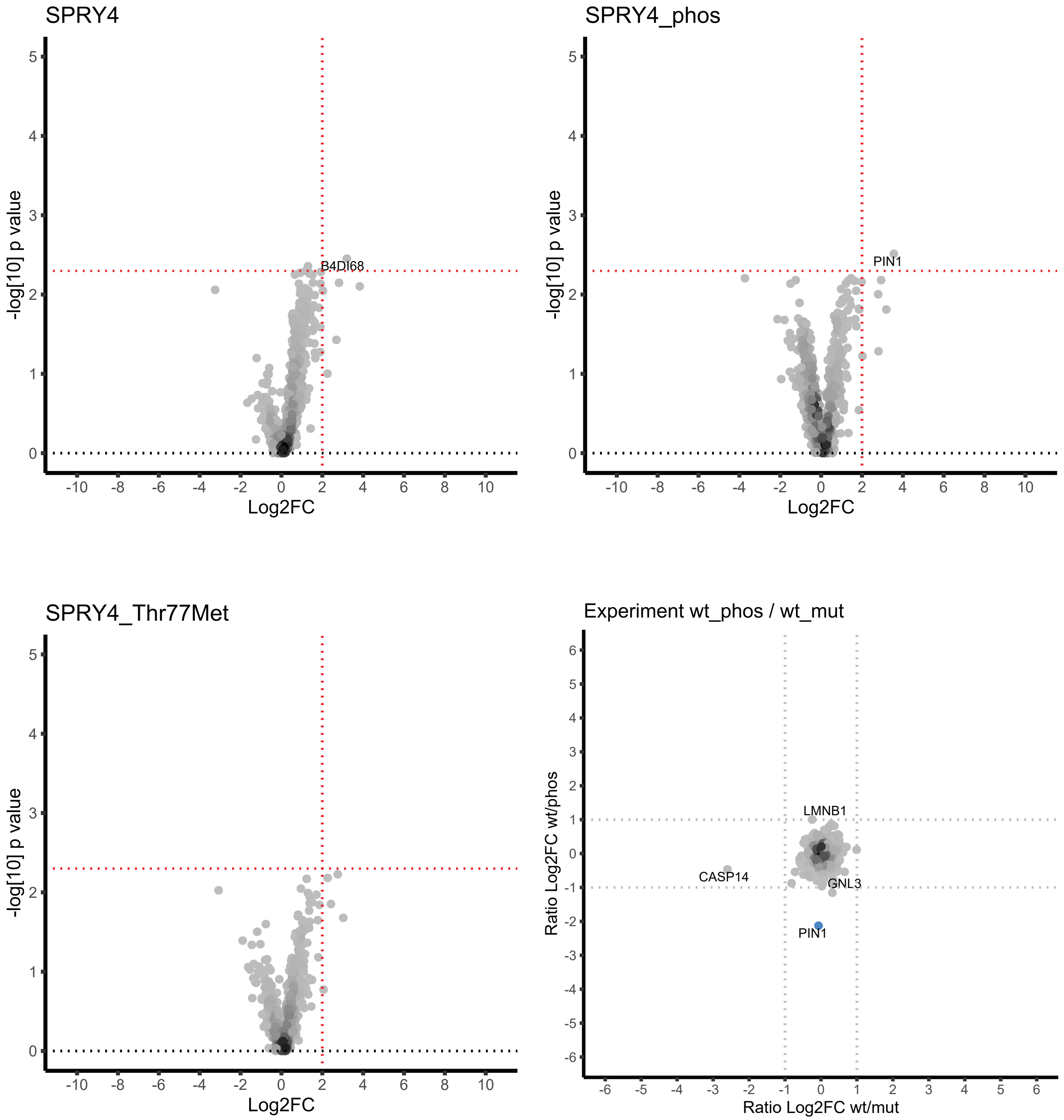

Supplementary Figure 32: Volcano and SILAC scatter plots for the SPRY4 peptide candidate: LFQ values were used to perform a Wilcoxon test where proteins identified in one peptide form (triplicates) were compared to all the proteins identified in all other WT and Mutant peptide forms. The calculated p-values and fold changes (Significance:Log2Fold change > 2 and p-value < 0.005) were plotted as Volcano plots (Top and bottom right). The log2 transformed SILAC ratios were plotted on scatter plots with wt/mut ratios on the x-axis and wt/phos ratios on the y-axis (bottom left) whereas the phos/mut ratios are not shown. SILAC ratios reveal differential interactors. To point out LFQ specific interactors in the scatter plots we used colored nodes as follows: Blue; phospho-peptide specific, yellow; wt-peptide specific, red; mut-peptide specific, orange; wt and mut-peptide specific, green; wt and phospho-peptide specific, purple; mut and phospho-peptide specific, and pink; wt, mut and phospho-peptide specific.

SRRM1: APEKKEKpTPELPEPS

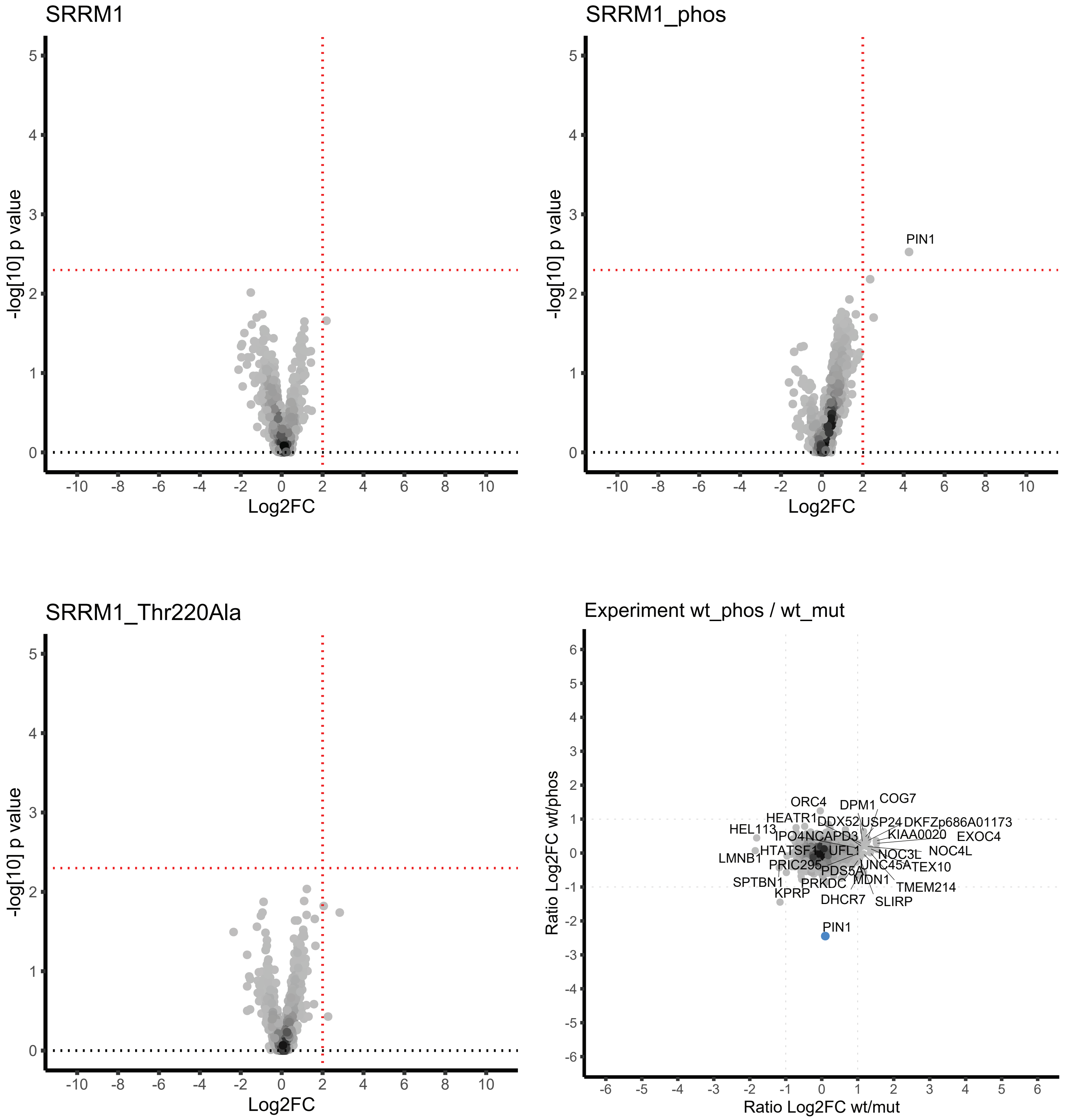

Supplementary Figure 33: Volcano and SILAC scatter plots for the SRRM1 peptide candidate: LFQ values were used to perform a Wilcoxon test where proteins identified in one peptide form (triplicates) were compared to all the proteins identified in all other WT and Mutant peptide forms. The calculated p-values and fold changes (Significance:Log2Fold change > 2 and p-value < 0.005) were plotted as Volcano plots (Top and bottom right). The log2 transformed SILAC ratios were plotted on scatter plots with wt/mut ratios on the x-axis and wt/phos ratios on the y-axis (bottom left) whereas the phos/mut ratios are not shown. SILAC ratios reveal differential interactors. To point out LFQ specific interactors in the scatter plots we used colored nodes as follows: Blue; phospho-peptide specific, yellow; wt-peptide specific, red; mut-peptide specific, orange; wt and mut-peptide specific, green; wt and phospho-peptide specific, purple; mut and phospho-peptide specific, and pink; wt, mut and phospho-peptide specific.

STXBP5L: QHIPGPGpSIEGMKGA

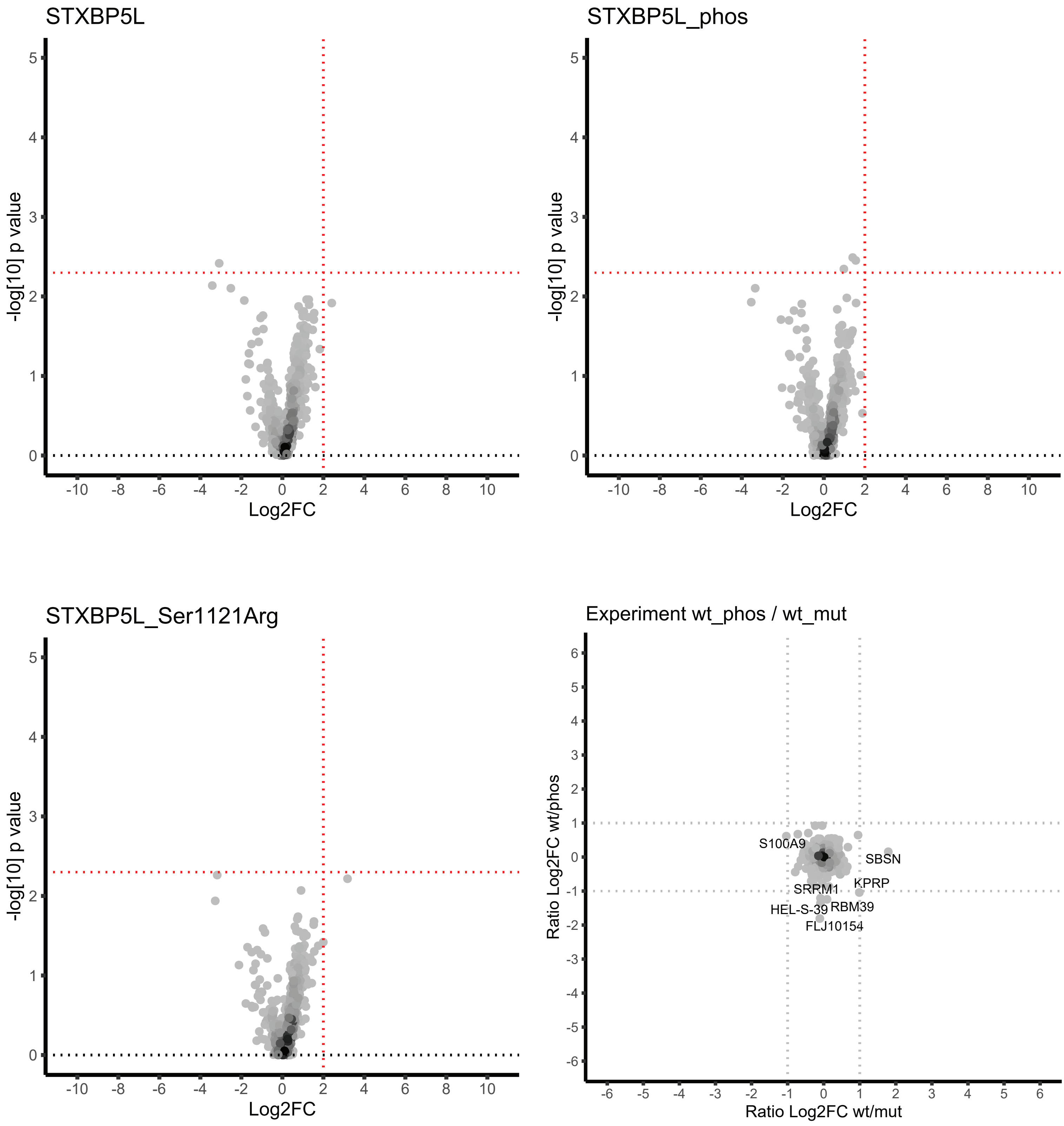

Supplementary Figure 34: Volcano and SILAC scatter plots for the STXBP5L peptide candidate: LFQ values were used to perform a Wilcoxon test where proteins identified in one peptide form (triplicates) were compared to all the proteins identified in all other WT and Mutant peptide forms. The calculated p-values and fold changes (Significance:Log2Fold change > 2 and p-value < 0.005) were plotted as Volcano plots (Top and bottom right). The log2 transformed SILAC ratios were plotted on scatter plots with wt/mut ratios on the x-axis and wt/phos ratios on the y-axis (bottom left) whereas the phos/mut ratios are not shown. SILAC ratios reveal differential interactors. To point out LFQ specific interactors in the scatter plots we used colored nodes as follows: Blue; phospho-peptide specific, yellow; wt-peptide specific, red; mut-peptide specific, orange; wt and mut-peptide specific, green; wt and phospho-peptide specific, purple; mut and phospho-peptide specific, and pink; wt, mut and phospho-peptide specific.

SYNRG: QETPNECpSDDFGEFQ

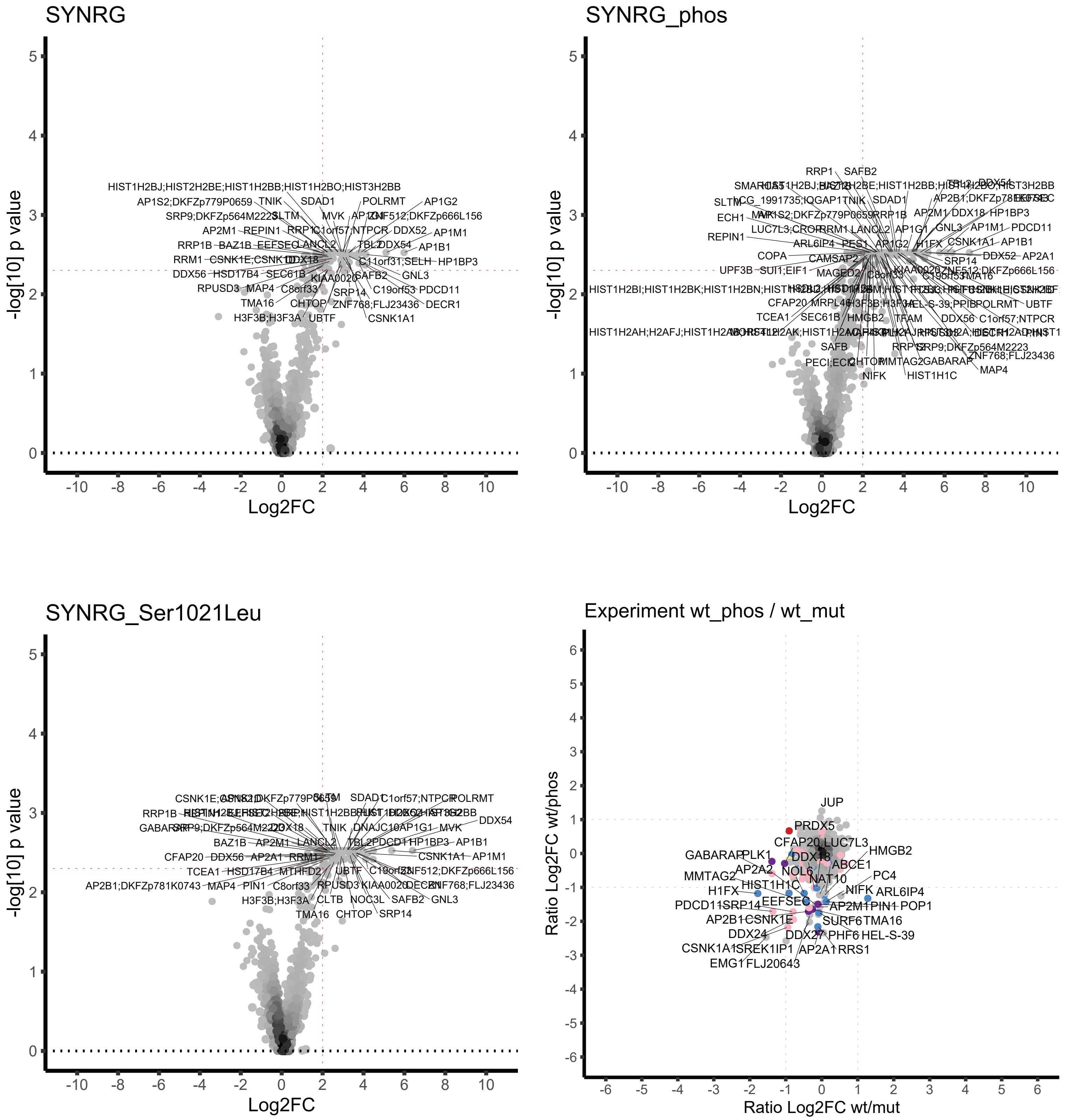

Supplementary Figure 35: Volcano and SILAC scatter plots for the SYNRG peptide candidate: LFQ values were used to perform a Wilcoxon test where proteins identified in one peptide form (triplicates) were compared to all the proteins identified in all other WT and Mutant peptide forms. The calculated p-values and fold changes (Significance:Log2Fold change > 2 and p-value < 0.005) were plotted as Volcano plots (Top and bottom right). The log2 transformed SILAC ratios were plotted on scatter plots with wt/mut ratios on the x-axis and wt/phos ratios on the y-axis (bottom left) whereas the phos/mut ratios are not shown. SILAC ratios reveal differential interactors. To point out LFQ specific interactors in the scatter plots we used colored nodes as follows: Blue; phospho-peptide specific, yellow; wt-peptide specific, red; mut-peptide specific, orange; wt and mut-peptide specific, green; wt and phospho-peptide specific, purple; mut and phospho-peptide specific, and pink; wt, mut and phospho-peptide specific.

TNNT2: ARKKKALpSNMMHFGG

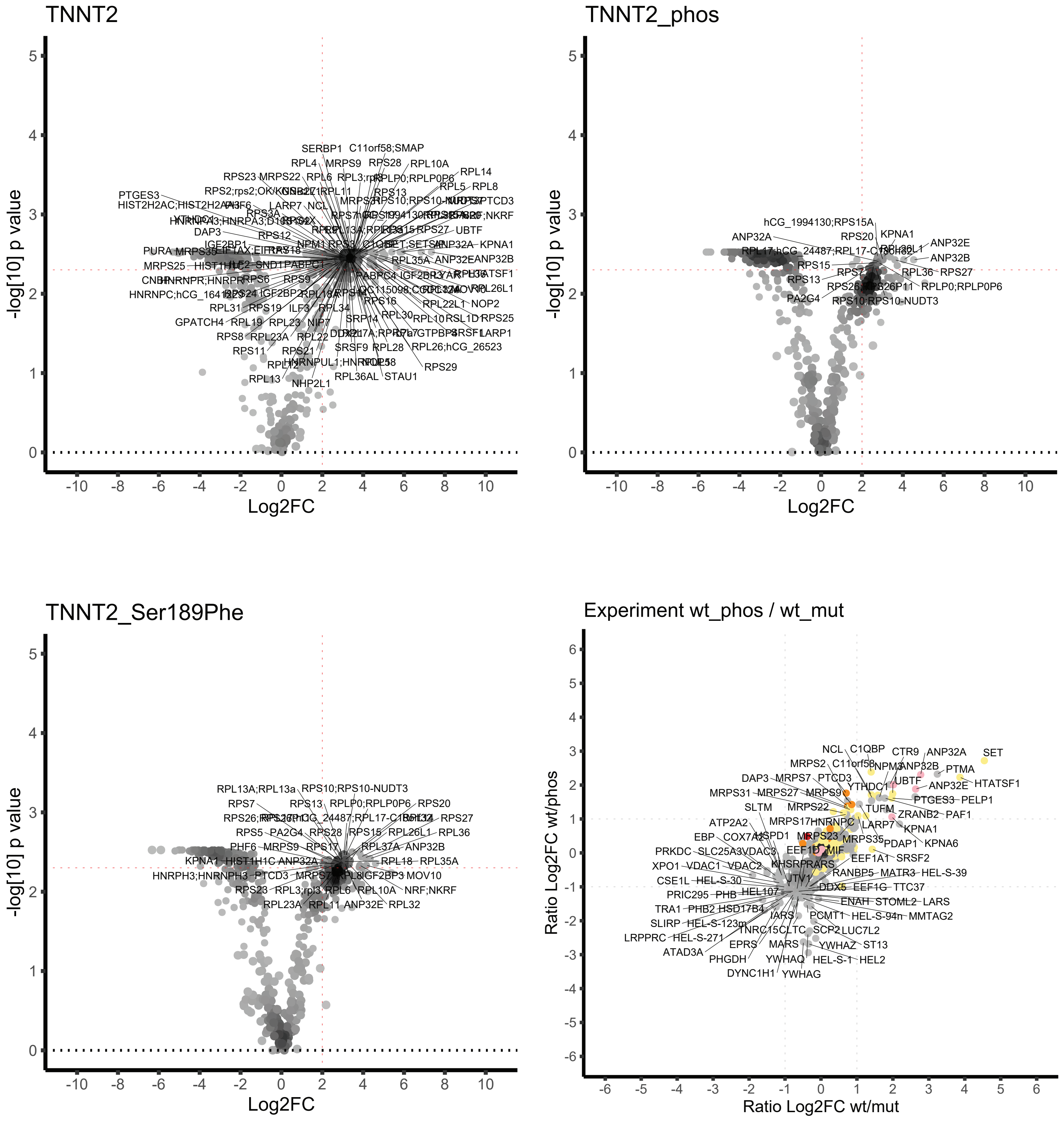

Supplementary Figure 36: Volcano and SILAC scatter plots for the TNNT2 peptide candidate: LFQ values were used to perform a Wilcoxon test where proteins identified in one peptide form (triplicates) were compared to all the proteins identified in all other WT and Mutant peptide forms. The calculated p-values and fold changes (Significance:Log2Fold change > 2 and p-value < 0.005) were plotted as Volcano plots (Top and bottom right). The log2 transformed SILAC ratios were plotted on scatter plots with wt/mut ratios on the x-axis and wt/phos ratios on the y-axis (bottom left) whereas the phos/mut ratios are not shown. SILAC ratios reveal differential interactors. To point out LFQ specific interactors in the scatter plots we used colored nodes as follows: Blue; phospho-peptide specific, yellow; wt-peptide specific, red; mut-peptide specific, orange; wt and mut-peptide specific, green; wt and phospho-peptide specific, purple; mut and phospho-peptide specific, and pink; wt, mut and phospho-peptide specific.

TTC7A: PDAHDADpSGSRRASS

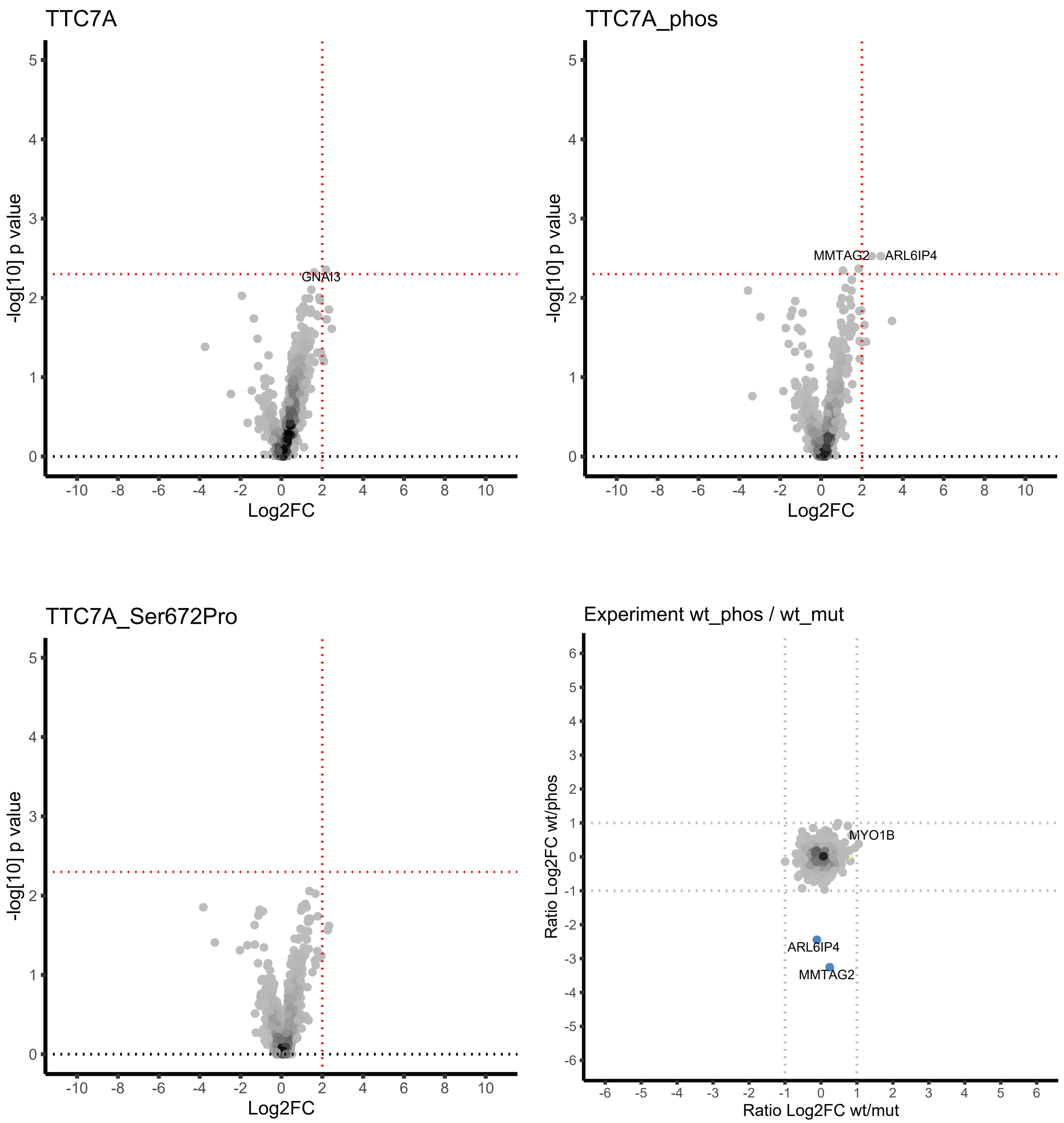

Supplementary Figure 37: Volcano and SILAC scatter plots for the TTC7A peptide candidate: LFQ values were used to perform a Wilcoxon test where proteins identified in one peptide form (triplicates) were compared to all the proteins identified in all other WT and Mutant peptide forms. The calculated p-values and fold changes (Significance:Log2Fold change > 2 and p-value < 0.005) were plotted as Volcano plots (Top and bottom right). The log2 transformed SILAC ratios were plotted on scatter plots with wt/mut ratios on the x-axis and wt/phos ratios on the y-axis (bottom left) whereas the phos/mut ratios are not shown. SILAC ratios reveal differential interactors. To point out LFQ specific interactors in the scatter plots we used colored nodes as follows: Blue; phospho-peptide specific, yellow; wt-peptide specific, red; mut-peptide specific, orange; wt and mut-peptide specific, green; wt and phospho-peptide specific, purple; mut and phospho-peptide specific, and pink; wt, mut and phospho-peptide specific.

TTF1: TLAMPEGpSQAGREAG

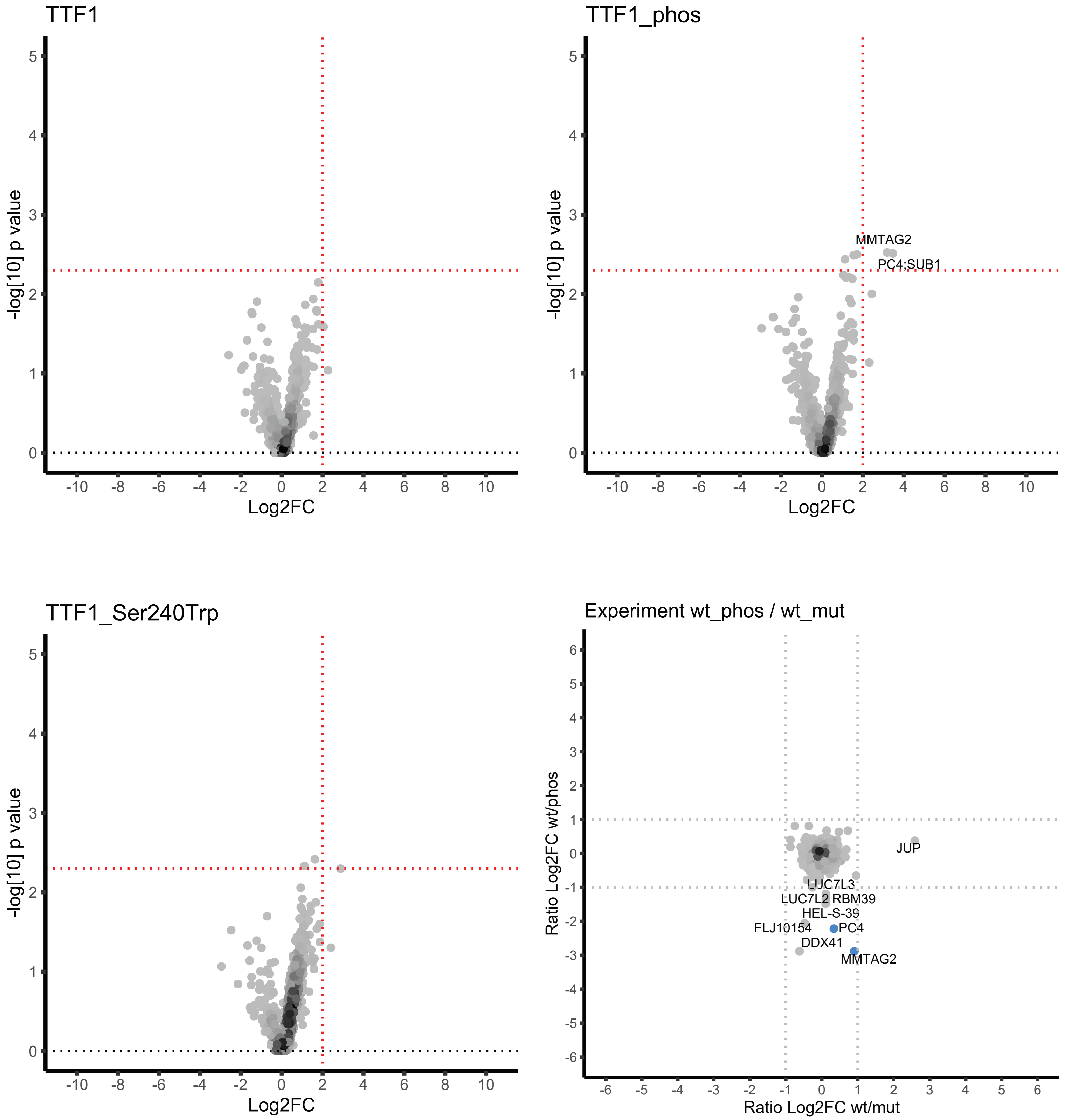

Supplementary Figure 38: Volcano and SILAC scatter plots for the TTF1 peptide candidate: LFQ values were used to perform a Wilcoxon test where proteins identified in one peptide form (triplicates) were compared to all the proteins identified in all other WT and Mutant peptide forms. The calculated p-values and fold changes (Significance:Log2Fold change > 2 and p-value < 0.005) were plotted as Volcano plots (Top and bottom right). The log2 transformed SILAC ratios were plotted on scatter plots with wt/mut ratios on the x-axis and wt/phos ratios on the y-axis (bottom left) whereas the phos/mut ratios are not shown. SILAC ratios reveal differential interactors. To point out LFQ specific interactors in the scatter plots we used colored nodes as follows: Blue; phospho-peptide specific, yellow; wt-peptide specific, red; mut-peptide specific, orange; wt and mut-peptide specific, green; wt and phospho-peptide specific, purple; mut and phospho-peptide specific, and pink; wt, mut and phospho-peptide specific.

TTR: ASGKTSEpSGELHGLT

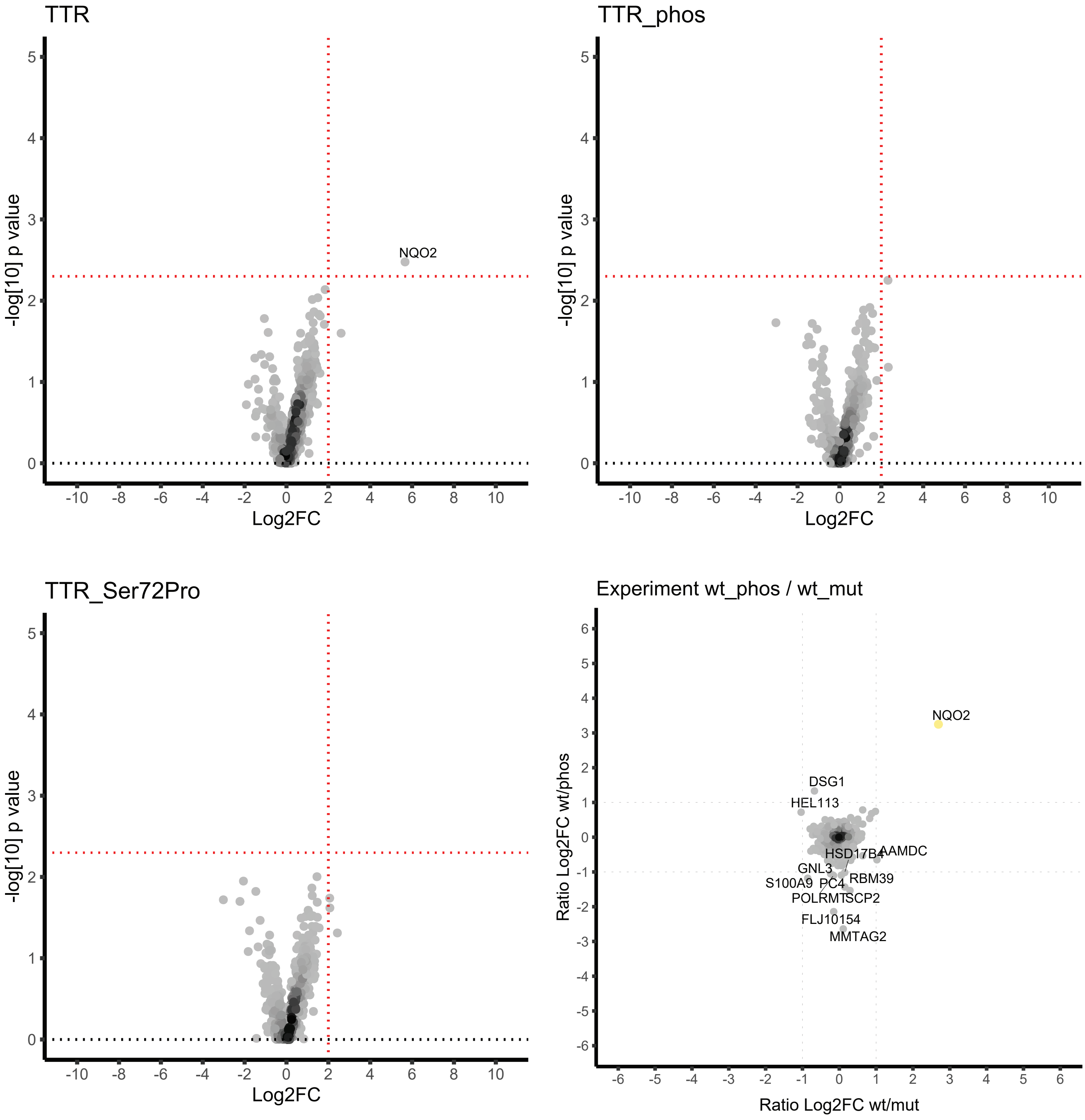

Supplementary Figure 39: Volcano and SILAC scatter plots for the TTR peptide candidate: LFQ values were used to perform a Wilcoxon test where proteins identified in one peptide form (triplicates) were compared to all the proteins identified in all other WT and Mutant peptide forms. The calculated p-values and fold changes (Significance:Log2Fold change > 2 and p-value < 0.005) were plotted as Volcano plots (Top and bottom right). The log2 transformed SILAC ratios were plotted on scatter plots with wt/mut ratios on the x-axis and wt/phos ratios on the y-axis (bottom left) whereas the phos/mut ratios are not shown. SILAC ratios reveal differential interactors. To point out LFQ specific interactors in the scatter plots we used colored nodes as follows: Blue; phospho-peptide specific, yellow; wt-peptide specific, red; mut-peptide specific, orange; wt and mut-peptide specific, green; wt and phospho-peptide specific, purple; mut and phospho-peptide specific, and pink; wt, mut and phospho-peptide specific.

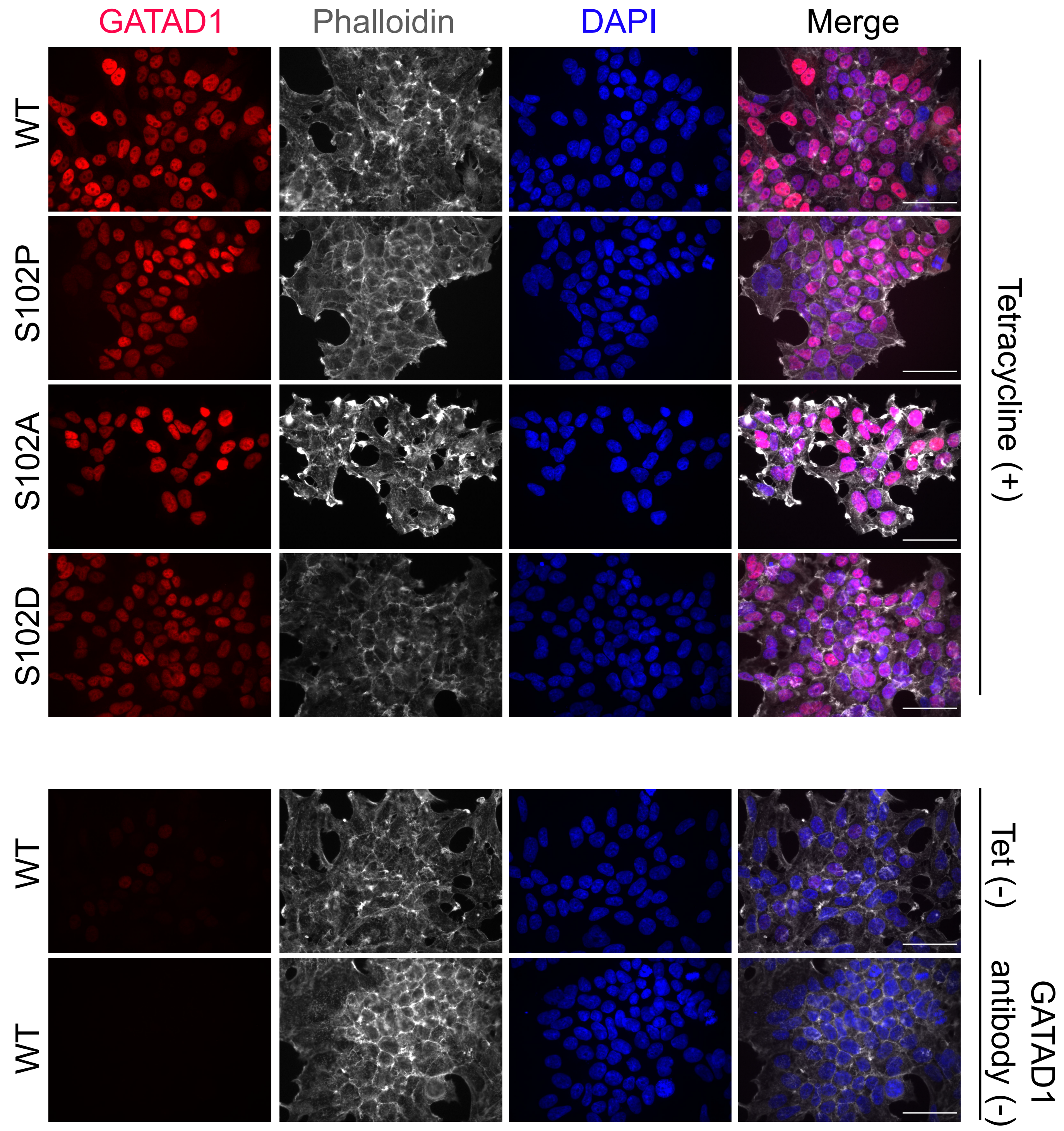

Supplementary Figure 40: Immunofluorescence images of FlpIn-293 cells that express BirA*-FLAG-GATAD1 in a tetracycline inducible manner. The images display a nuclear localization of GATAD1 WT protein as expected, indicating that the BirA* and FLAG do not alter protein localization (Top lane). Similarly, all GATAD1 variants (S102P, S102D, and S102A) also localize predominantly in the nucleus. Implying that the mutation does not perturb the nuclear localization of the protein.

We observed little to no leakage of expression in the absence of 1 μg/μl of tetracycline and no signal in the absence of primary GATAD1 antibody (Bottom two lanes).Scale bar = 50 μm

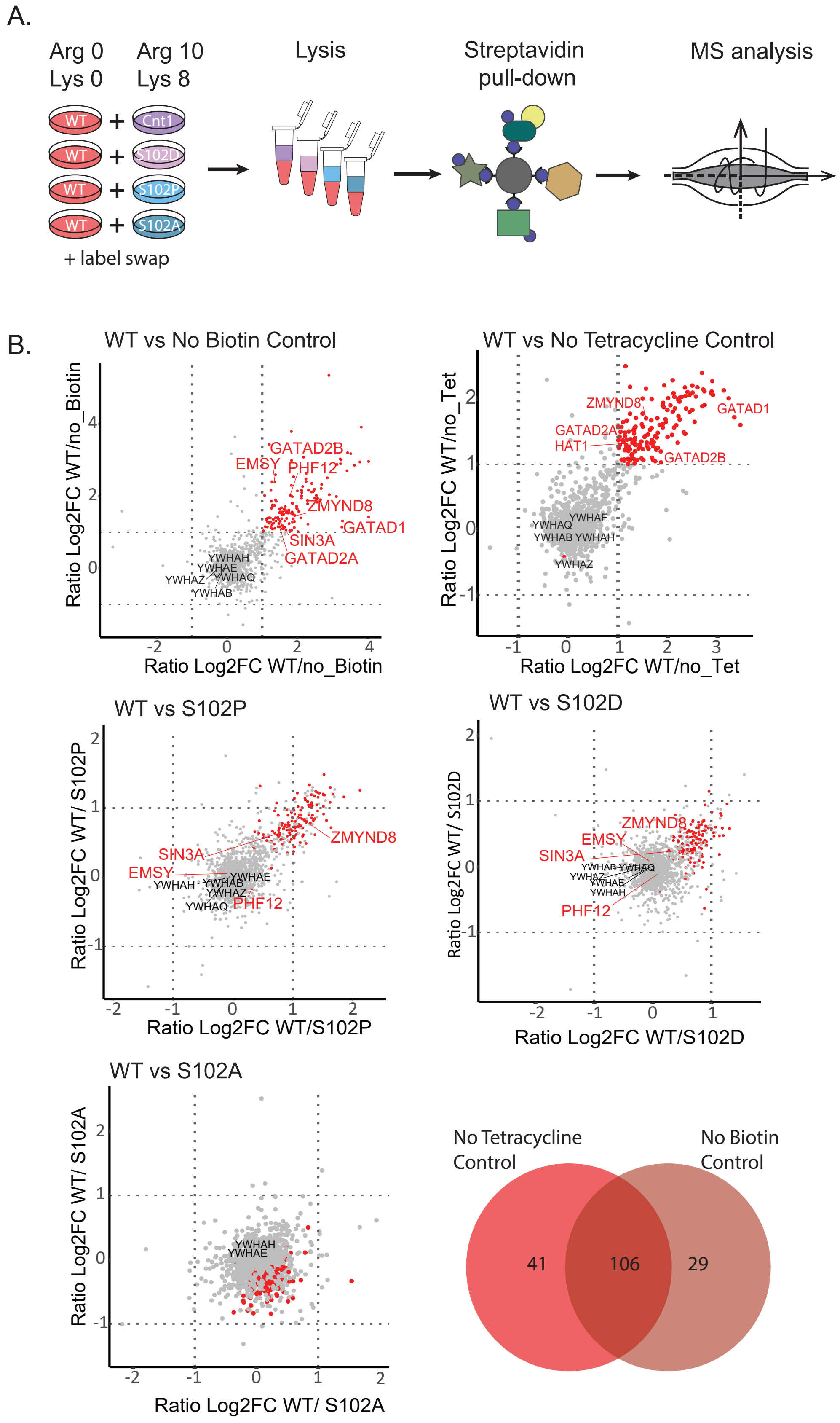

Supplementary Figure 41: BioID results for full-length GATAD1. A) Experimental design. B) Scatter plots display protein SILAC ratios of two biological replicates (forward and reverse) for different experiments. Top left: WT GATAD1 versus the "no biotin" control identifies several well-established GATAD1 interactors (red and labeled) and 130 additional proteins (red), indicating that the BioID data reflects GATAD1 biology. Top right: WT GATAD1 versus the "no tetracycline" control. There is a high similarity between the no biotin and no tetracycline controls with 106 proteins shared among the two (Pie chart at the bottom). In the other three scatter plots, WT GATAD1 is compared to different mutants. The S102P and S102D mutant show similar but overall somewhat reduced interactions while the S102A mutant is essentially identical to the wild-type. 14-3-3 family proteins were detected (YWHAB, YWHAE, YWHAH, YWHAQ, and YWHAZ) but do not show any specific interaction with any of the GATAD1 proteins

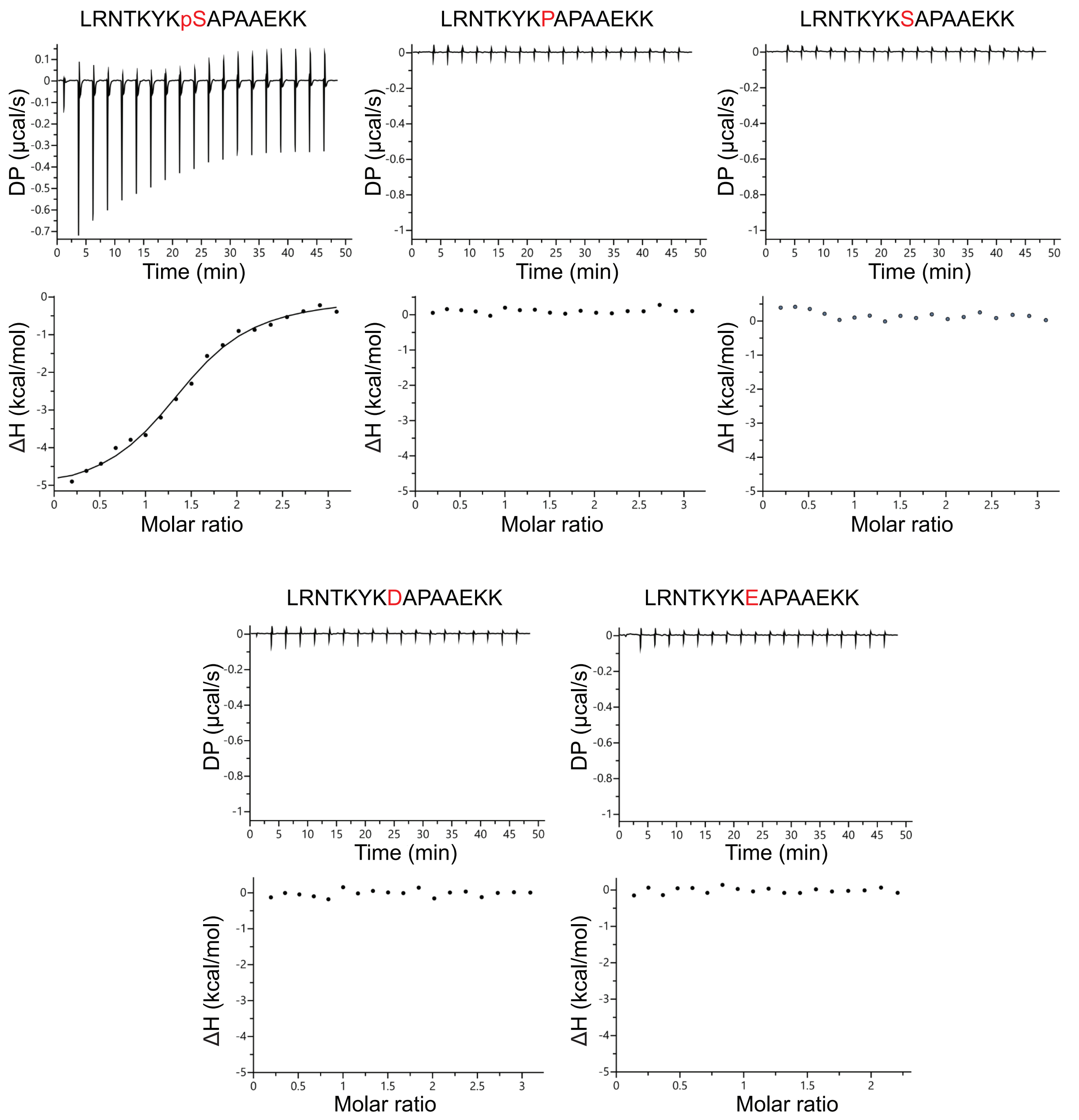

Supplementary Figure 42: Titration data of the ITC experiment. For each peptide form, on top is the simulated ITC raw data plot, and below is the fitted ITC binding curve.

Supplementary Table 1: Data collection and refinement statistics

	14-3-3ε : GATAD1 peptide
**Data collection**	
Space group	R32
Cell dimensions	
a, *b*, *c* (Å)	123.8, 123.8, 250.2,
α, β, γ (°)	90.0, 90.0, 120.0
Resolution (Å)*	49.70-3.23 (3.42-3.23)
*Rmeas**	35.0 (296.0)
< *I* / σ(*I*) >*	6.9 (0.8)
Completeness (%)*	99.9 (99.9)
Redundancy*	10.4 (10.6)
CC (1/2)	99.5 (36.8)
**Refinement**	
Resolution (Å)	3.23
No. reflections	11549
*R*work/ *R*free	0.230/0.279
No. atoms	
Protein	3926
Ion	2
Water	12
Mean B factor (Å2)	122.9
R.m.s deviations	
Bond lengths (Å)	0.001
Bond angles (º)	1.045

* Data in highest resolution shell are indicated in parenthesis.
