# Supplementary material for: Pathogenic mutations of human phosphorylation sites affect protein–protein interactions
Source: Nat Commun. 2024 Apr 11;15:3146. doi: 10.1038/s41467-024-46794-8 (PMC11009412; doi:10.1038/s41467-024-46794-8)
Supplement: Supplementary file 3 — Description of Additional Supplementary Files [file 41467_2024_46794_MOESM3_ESM.pdf]

Description of Additional Supplementary Files

File Name: Supplementary Data 1
Description: Disease candidate and peptide selection. This data table contains the mutation candidates studied in this paper and the diseases they cause.

File Name: Supplementary Data 2
Description: Specific and differential peptide-protein interactions. This data table contains information on all the proteins identified as significant and differential interactors after performing the PRISMA.

File Name: Supplementary Data 3
Description: SLiMs in candidate peptides. Data information on the SLiM-Domain pairs identified in the PRISMA.

File Name: Supplementary Data 4
Description: Proteomic data for FLAG IPs. Data from the FLAG IP experiments. It contains the MaxQuant output protein groups and all the subsequent filtering and analysis.

File Name: Supplementary Data 5
Description: Proteomic data for alanine scanning. This data table contains information from MaxQuant output protein groups and subsequent filtering and analysis.

File Name: Supplementary Data 6
Description: Proteomic data for BioID. The data table contains the results of the BioID experiment, including the MaxQuant output and the subsequent data filtering and transformation.
