# Supplementary material for: Pathogenic mutations of human phosphorylation sites affect protein–protein interactions
Source: Nat Commun. 2024 Apr 11;15:3146. doi: 10.1038/s41467-024-46794-8 (PMC11009412; doi:10.1038/s41467-024-46794-8)
Supplement: Supplementary file 10 — Reporting Summary [file 41467_2024_46794_MOESM10_ESM.pdf]

nature portfolio

Corresponding author(s): Matthias Selbach

Last updated by author(s): Feb 23, 2024

Reporting Summary

Nature Portfolio wishes to improve the reproducibility of the work that we publish. This form provides structure for consistency and transparency in reporting. For further information on Nature Portfolio policies, see our Editorial Policies and the Editorial Policy Checklist.

Statistics

For all statistical analyses, confirm that the following items are present in the figure legend, table legend, main text, or Methods section.

n/a	Confirmed	
☐	☒	The exact sample size (*n*) for each experimental group/condition, given as a discrete number and unit of measurement
☐	☒	A statement on whether measurements were taken from distinct samples or whether the same sample was measured repeatedly
☐	☒	The statistical test(s) used AND whether they are one- or two-sided<br>*Only common tests should be described solely by name; describe more complex techniques in the Methods section.*
☒	☐	A description of all covariates tested
☐	☒	A description of any assumptions or corrections, such as tests of normality and adjustment for multiple comparisons
☒	☐	A full description of the statistical parameters including central tendency (e.g. means) or other basic estimates (e.g. regression coefficient) AND variation (e.g. standard deviation) or associated estimates of uncertainty (e.g. confidence intervals)
☐	☒	For null hypothesis testing, the test statistic (e.g. *F*, *t*, *r*) with confidence intervals, effect sizes, degrees of freedom and *P* value noted<br>*Give P values as exact values whenever suitable.*
☒	☐	For Bayesian analysis, information on the choice of priors and Markov chain Monte Carlo settings
☒	☐	For hierarchical and complex designs, identification of the appropriate level for tests and full reporting of outcomes
☒	☐	Estimates of effect sizes (e.g. Cohen's *d*, Pearson's *r*), indicating how they were calculated

Our web collection on statistics for biologists contains articles on many of the points above.

Software and code

Policy information about availability of computer code

Data collection	No specific code was used for data selection.
Data analysis	For PRISMA experiment, mass spectrometry MaxQuant version 1.6.2.6a and R (R version 4.2.1 and Rstudio version 2022.07.1) were used. The peptide-protein network was generated on Cytoscape v.3.9.1<br>The code used to analyse the MaxQuant outputs has been uploaded to github repository [https://github.com/Trruste/PRISMA-phosphoarray/blob/main/PRISMA_script.Rmd]<br>SLiM analysis is available on [https://github.com/BIMSBbioinfo/collab_rrustemi_selbach_prisma].<br>Mass spectrometry raw files of the BioID proximity labelling experiment were analysed using MaxQuant, version 2.0.3.0. and R (R version 4.2.1 and Rstudio version 2022.07.1)<br>Mass spectrometry raw files form the AP-MS interactome studies were analysed with MaxQuant version 1.6.7.0. Subsequent analysis was performed using R (R version 4.2.1 and Rstudio version 2022.07.1).<br>Mass spectrometry raw files from the Alanine scanning experiment were analysed with MaxQuant (version 1.6.3.4). Subsequent analysis was performed using Perseus (version 1.6.7.0).

For manuscripts utilizing custom algorithms or software that are central to the research but not yet described in published literature, software must be made available to editors and reviewers. We strongly encourage code deposition in a community repository (e.g. GitHub). See the Nature Portfolio guidelines for submitting code & software for further information.

Data

Policy information about availability of data

All manuscripts must include a data availability statement. This statement should provide the following information, where applicable:

- Accession codes, unique identifiers, or web links for publicly available datasets
- A description of any restrictions on data availability
- For clinical datasets or third party data, please ensure that the statement adheres to our policy

Data Availability
Mass spectrometry raw files have been deposited to the ProteomeXchange Consortium via the PRIDE (Vizcaíno et al. 2015) partner repository. The accession codes for the uploaded data are as follows: PRISMA screen; PXD043787 [http://www.ebi.ac.uk/pride/archive/projects/PXD043787], AP-MS interactome studies; PXD046950 [http://www.ebi.ac.uk/pride/archive/projects/PXD046950], BioID-proximity labeling; PXD043789 [http://www.ebi.ac.uk/pride/archive/projects/PXD043789] and Alanine Scanning: PXD043788 [http://www.ebi.ac.uk/pride/archive/projects/PXD043788]. The processed mass spectrometry data is provided in the supplementary files. The 14-3-3ε with the GATAD1 95-LRNTKYKpSAPAAEKK-109 peptide structure has been submitted to the Protein Data Bank under the code 8Q1S [http://doi.org/10.2210/pdb8Q1S/pdb]. Raw data used for the quantification plots in figure 6C and D is provided as source data.

Code availability
The code used to analyze the PRISMA data is available on https://github.com/Trruste/PRISMA-phosphoarray/blob/main/PRISMA_script.Rmd and the code for a more detailed SLiM analysis is available on https://github.com/BIMSBbioinfo/collab_rrustemi_selbach_prisma.

Human research participants

Policy information about studies involving human research participants and Sex and Gender in Research.

Reporting on sex and gender	NA
Population characteristics	NA
Recruitment	NA
Ethics oversight	NA

Note that full information on the approval of the study protocol must also be provided in the manuscript.

Field-specific reporting

Please select the one below that is the best fit for your research. If you are not sure, read the appropriate sections before making your selection.

☒ Life sciences ☐ Behavioural & social sciences ☐ Ecological, evolutionary & environmental sciences

For a reference copy of the document with all sections, see nature.com/documents/nr-reporting-summary-flat.pdf

Life sciences study design

All studies must disclose on these points even when the disclosure is negative.

Sample size	The sample size for the PRISMA screen was limited to the starting database, PTMVar, and the filtering strategy. For all other experiments we picked a standard n=3 biological replicates
Data exclusions	Two of the peptide candidates were mixed up during the sample multiplexing, therefore, were excluded from statistical analysis.
Replication	The experiments were performed in biological triplicates to ensure that only the reproducible results are considered.
Randomization	Randomization is not relevant because internal controls were used to identify differential binders (SILAC control was used)
Blinding	Blinding is not relevant because data acquisition and analysis was performed in an automated way.

Reporting for specific materials, systems and methods

We require information from authors about some types of materials, experimental systems and methods used in many studies. Here, indicate whether each material, system or method listed is relevant to your study. If you are not sure if a list item applies to your research, read the appropriate section before selecting a response.

Materials & experimental systems

n/a	Involved in the study	
☐	☒	Antibodies
☐	☒	Eukaryotic cell lines
☒	☐	Palaeontology and archaeology
☒	☐	Animals and other organisms
☒	☐	Clinical data
☒	☐	Dual use research of concern

Methods

n/a	Involved in the study	
☒	☐	ChIP-seq
☒	☐	Flow cytometry
☒	☐	MRI-based neuroimaging

Antibodies

Antibodies used

mouse monoclonal IgG anti-GATAD1 (sc-81092, Dilution 1:100, Provider: Santa Cruz),
goat anti-mouse IgG (H+L) Alexa Fluor 488 (A11001, Dilution 1:500, Provider: Invitrogen),
phalloidin - Alexa 594 (A12381, Dilution 1:500, Provider: Invitrogen)

Validation

GATAD1(GATA9A1D3) is a mouse monoclonal antibody raised against a recombinant protein corresponding to the C-terminal region of human origin
Product Citation: Varier, RA. et al. 2016. The Journal of biological chemistry. 291: 7313-24.

Eukaryotic cell lines

Policy information about cell lines and Sex and Gender in Research

Cell line source(s)

EK-293 (DSMZ Cat#ACC635), HEK-293T (DSMZ Cat#ACC305), and Flp-InTM-293 T-REx (Thermo Fisher Scientific, R78007)

Authentication

None of the cell lines were authenticated.

Mycoplasma contamination

Cell lines used in this paper all tested negative for mycoplasma (HEK-293, HEK-293T, Flp-In-293 T-REx)

Commonly misidentified lines
(See ICLAC register)

No misidentified cell lines were used
